# Three-Carbon Linked Dihydroartemisinin-Isatin Hybrids: Design, Synthesis and Their Antiproliferative Anticancer Activity

**DOI:** 10.3389/fphar.2022.834317

**Published:** 2022-01-26

**Authors:** Min Dong, Guili Zheng, Feng Gao, Min Li, Chen Zhong

**Affiliations:** ^1^ Department of Oncology, No. 960 Hospital of PLA, Jinan, China; ^2^ Key Laboratory for Experimental Teratology of the Ministry of Education and Center for Experimental Nuclear Medicine, School of Basic Medical Sciences, Cheeloo College of Medicine, Shandong University, Jinan, China; ^3^ Department of Nuclear Medicine, No. 960 Hospital of PLA, Jinan, China

**Keywords:** dihydroartemisinin, hybrid molecules, multidrug resistance, structure-activity relationship, anticancer

## Abstract

Fifteen dihydroartemisinin-isatin hybrids (**5a-e** and **6a-j)** linked with three-carbon were designed, synthesized. The antiproliferative activity against lung cancer cell lines including drug-sensitive A549, doxorubicin-resistant A549 (A549/DOX) and cisplatin-resistant A549 (A549/DDP) lung cancer cell lines was tested. The cytotocivity towards normal lung epithelial BEAS-2B cell line was also investigated. From the structure-activity relationship (SAR), it was found that hydrogen bond donors (especially hydroxime and thiosemicarbazide) at C-3 position and electron-withdrawing groups (fluoro and chloro) at C-5 position of isatin moiety were beneficial for the activity. A significant part of them (half maximal inhibitory concentration/IC_50_: 5.72–55.52 *μ*M) demonstrated considerable antiproliferative activity, and the activity was superior to that of dihydroartemisinin (IC_50_: 69.42–88.03 *μ*M) and artemisinin (IC_50_: >100 *μ*M). In particular, two hybrids **6a, e** (IC_50_: 5.72–9.84 *μ*M) were not inferior to doxorubicin (IC_50_: 4.06 *μ*M) and cisplatin (IC_50_: 9.38 *μ*M) against drug-sensitive A549 cells and were more potent than doxorubicin (IC_50_: 54.32 and 15.10 *μ*M) and cisplatin (IC_50_: 19.74 and 66.89 *μ*M) against multidrug-resistant A549/DOX and A549/DDP lung cancer cell lines. In addition, hybrids **6a, e** (IC_50_: >100 *μ*M) showed no toxicity towards BEAS-2B cells, proving their excellent selectivity profile. Furthermore, hybrid **6a** also possessed good stability in mouse and human microsomes, as well as excellent pharmacokinetic properties. Accordingly, hybrid **6a** could serve as a promising anti-lung cancer chemotherapeutic candidate for further preclinical evaluations.

## Introduction

Lung cancer, caused by several environmental and genetic variables (Zhang et al., 2021; Yang et al., 2022), is the leading cause of cancer related deaths and is responsible for around 20% of all cancer deaths with an estimated 1.8 million new cases and 1.6 million deaths annually (Hirsch et al., 2017; Kramer and Annema, 2021). According to the histopathological characteristics, lung cancer is mainly divided into non-small cell lung cancer (NSCLC) and small cell lung cancer (SCLC), and NSCLC accounts for about 80–85% of lung cancers (Sławiński et al., 2020; Xie et al., 2021). Despite the advances in cancer diagnosis and therapy, the advent of chemotherapeutic resistance in lung cancer and serious side effects are the major obstacles for the effective treatment (Bade and Dela Cruz, 2020; Coakley and Popat, 2020). Therefore, it is imperative to develop novel anti-lung cancer agents.

**FIGURE 1:**
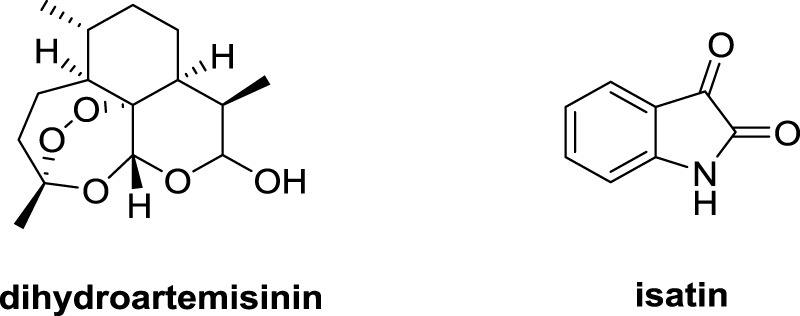
Chemical structures of dihydroartemisinin and isatin.

Dihydroartemisinin (DHA, Figure 1) has a unique sesquiterpene endoperoxide lactone moiety and could form highly reactive free radicals in the presence of ferrous ion (Fe^II^). The concentration of Fe^II^ in cancer cells is 1,000 times higher than that in normal cells (Dai et al., 2017; Kiani et al., 2020). DHA could interact with Fe^II^, resulting in an obvious inhibitory effect on lung cancer cells including lung squamous carcinoma, lung adenocarcinoma and large cells lung carcinoma without significant cytotoxicity to normal cells (Gao et al., 2020; Li et al., 2021). Isatin derivatives displayed promising anti-lung cancer activity, and the isatin-based nintedanib plus docetaxel has already been approved for treatment of patients with advanced NSCLC after first-line chemotherapy in Europe (Ding et al., 2020; Hou et al., 2020). Hence, DHA and isatin may provide useful templates for the development of novel anti-lung cancer agents.

Hybrids synthesized by fusing or conjugating different active pharmacophores together are capable to modulate multiple targets, consequently enhancing the efficacy, overcoming drug resistance and reducing side effects (Nepali et al., 2014; Sharma et al., 2014; Mishra and Singh, 2016; Choudhary et al., 2018; Hou et al., 2021). Since both DHA and isatin hold potent anti-lung cancer activity, there is a high possibility that hybridization of DHA with isatin may provide novel anti-lung cancer candidates with high activity and efficacy.

**FIGURE 2:**
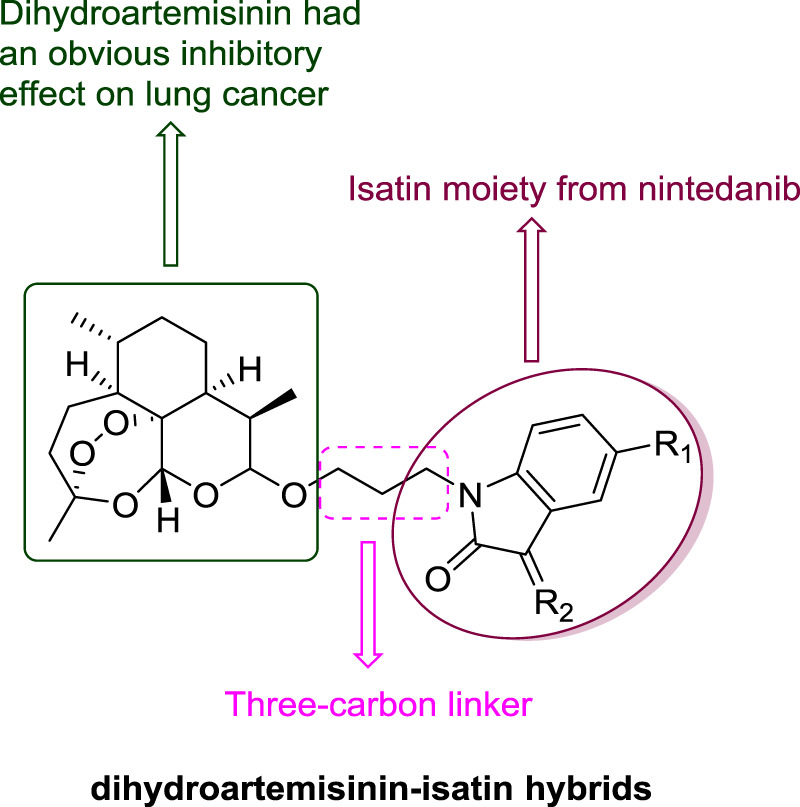
Chemical structures of dihydroartemisinin-isatin hybrids with three-carbon linker.

Compared with other linkers like thiazole linker, alkyl linkers are more flexible, probably making the hybrids with alkyl linkers much easier to bind with different targets. To evaluate the influence of the length of the alkyl linker between dihydroartemisinin and isatin, various of dihydroartemisinin-isatin hybrids with two-four-carbon linkers were designed and synthesized by our group. In this paper, we only designed and synthezed a series of dihydroartemisinin-isatin hybrids with three-carbon linker (Figure 2). Afterwards, the antiproliferative activity against drug-sensitive (A549), and drug-resistant [doxorubicin-resistant A549 (A549/DOX) and cisplatin-resistant A549 (A549/DDP)] lung cancer cell lines was evaluated. The cytotocivity towards normal lung epithelial cell line (BEAS-2B) was also investigated. Moreover, the liver stability and pharmacokinetic properties of the representative hybrids were also tested to search for the potential anti-lung cancer candidates for further studies. The biological activity and the pharmacokinetic properties of the hybrids with two carbon or four carbon linker are still under investigation, and the anticancer characteristics of the hybrids with different length of linkers will be reported in the near future.

## Results and Discussion

### Preparetion of the Desired Hybrids

**SCHEME 1:**
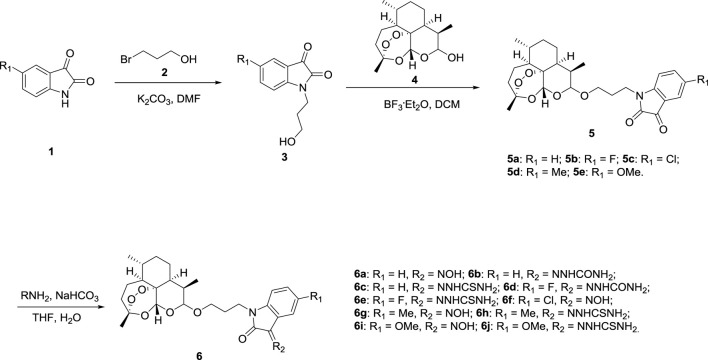
Synthesis of dihydroartemisinin-isatin hybrids **5a-e** and **6a-j**.

Scheme 1 describes the synthetic route of desired dihydroartemisinin-isatin hybrids **5a-e** and **6a-j**. (5-substituted) isatins **1** alkylating with 3-bromopropanol **2**, using potassium carbonate (K_2_CO_3_) as base yielded intermediates **3**. Intermediates **3** then reacted with dihydroartemisinin **4** in presence of boron trifluoride diethyl etherate (BF_3_^**.**^ OEt_2_), giving the desired dihydroartemisinin-isatin hybrids **5a-e**. Finally, the reation of hybrids **5a-e** with hydroxylamine/semicarbazide/thiosemicarbazide hydrochlorides using sodium bicarbonate (NaHCO_3_) as base provided the desired dihydroartemisinin-isatin hybrids **6a-j**. The chemical structures and yields of the desired hybrids were listed in Table 1. The structure of the desired dihydroartemisinin-isatin hybrids **5a-e** and **6a-j** were determined by HRMS, ^1^H NMR and ^13^C NMR. All target compounds should be a mixture of α- and β-configuration in dihydroartemisinin skeleton, and for hybrids **6a-j**, *E*- and *Z*-configuration should be existed at C-3 position of isatin moiety. Taking hybrid **6a** for an example, the ^1^H NMR (600 Hz, DMSO-*d*_6_) was as follows: δ 0.86–0.99 (m, 7H, H-15, H-16 and H-5a), 1.26–1.57 (m, 7H, H-5a_2_, H-7a_2_, H-6, H-14 and H-8a), 1.64–1.90 (m, 3H, H-7a_1_ and H-8), 1.95–2.06 (m, 3H, -CH_2_- linker and H-5a_1_), 2.25–2.66 (m, 3H, H-9, H-4a_1_ and H-4a_2_), 3.45–4.12 (m, 4H, -OCH_2_- linker and -NCH_2_- linker), 4.95 (d, *J* = 2.0 Hz, 1H, H-10), 5.47 (s, 1H, H-12), 6.88 (d, *J* = 4.0 Hz, 1H, isatin-H), 7.09 (t, *J* = 4.0 Hz, 1H, isatin-H), 7.36 (t, *J* = 4.0 Hz, 1H, isatin-H), 8.12 (d, *J* = 4.0 Hz, 1H), 11.04 (brs, 1H, NOH).

**TABLE 1:** Chemical structures and yields of dihydroartemisinin-isatin hybrids **5a-e** and **6a-j**.

Compd	R_1_	R_2_	Yield (%)
**5a**	H	O	61
**5b**	F	O	48
**5c**	Cl	O	53
**5d**	Me	O	57
**5e**	OMe	O	39
**6a**	H	NOH	87
**6b**	H	NNHCONH_2_	41
**6c**	H	NNHCSNH_2_	33
**6d**	F	NNHCONH_2_	48
**6e**	F	NNHCSNH_2_	26
**6f**	Cl	NOH	72
**6g**	Me	NOH	91
**6h**	Me	NNHCSNH_2_	30
**6i**	OMe	NOH	88
**6j**	OMe	NNHCSNH_2_	25

### Antiproliferative Activity and Cytotoxicity Study *in vitro*

3-(4,5-dimethylthiazol-2-yl)-2,5-diphenyltetrazolium bromide (MTT) assay was used to determined the antiproliferative activity of dihydroartemisinin-isatin hybrids **5a-e** and **6a-j** with three-carbon linker against A549, multidrug-resistant A549/DOX and A549/DDP lung cancer cell lines as well as cytotoxicity towards normal lung epithelial cell line (BEAS-2B). The inhibitory effects were expressed in terms of half maximal inhibitory concentration (IC_50_) values. It can be seen from Table 2 that nine desired dihydroartemisinin-isatin hybrids (**5c**, **6a**, **6c**, **6e-j,** IC_50_: 5.72–99.57 *µ*M) showed decent antiproliferative activity against A549, A549/DOX and A549/DDP lung cancer cell lines. These nine hybrids were much more active than artemisinin (IC_50_: >100 *µ*M), and seven of them (**6a**, **6c**, **6e-g**, **6i-j**) disaplayed superior inhibition to the parent drugs DHA (IC_50_: 69.42–88.03 *µ*M). The SAR revealed that introducing hydroxime and thiosemicarbazide into C-3 position of isatin moiety were beneficial for the activity, while carbonyl and semicarbazide had negative influence on the activity. Electron-withdrawing groups such as fluoro and chloro at C-5 position of isatin moiety could improve the activity to some extent, while electron-donating groups such as methyl and methoxy led to a significant loss of activity.

**TABLE 2:** *In vitro* antiproliferative activities, cytotoxicity of dihydroartemisinin-isatin hybrids **5a-e** and **6a-j**.

Compd	IC_50_ (*μ*M)				SI^a^	RI	
	A549	A549/DOX^b^	A549/DDP^c^	BEAS-2B		RI1^d^	RI2^e^
**5a**	>100	>100	>100	>100	-	-	-
**5b**	>100	>100	>100	>100	-	-	-
**5c**	53.57	49.73	79.81	>100	>1.8	0.93	1.49
**5d**	>100	>100	>100	>100	-	-	-
**5e**	>100	>100	>100	>100	-	-	-
**6a**	5.72	7.35	9.84	>100	>17.4	1.28	1.66
**6b**	>100	>100	>100	>100	-	-	-
**6c**	23.24	36.60	28.79	>100	>4.3	1.57	1.24
**6d**	>100	>100	>100	>100	-	-	-
**6e**	5.99	8.93	6.17	>100	>16.6	1.49	1.03
**6f**	24.74	26.88	31.95	>100	>4.0	1.08	1.29
**6g**	55.52	29.90	49.67	>100	>1.8	0.54	0.89
**6h**	99.57	87.31	94.23	>100	>1.0	0.88	0.95
**6i**	51.59	73.53	60.17	>100	>1.9	1.46	1.17
**6j**	52.62	78.46	40.68	>100	>1.9	1.68	0.77
Artemisinin	>100	>100	>100	>100	-	-	-
DHA^f^	69.42	88.03	75.91	>100	>1.4	1.27	1.09
cisplatin	9.38	19.74	66.89	89.63	9.5	2.10	7.13
doxorubicin	4.06	54.32	15.10	93.76	23.1	13.38	3.72

^a^Selectivity index: IC_50(BEAS-2B)_/IC_50(A549)_.

b Doxorubicin-resistant A549 cells.

c Cisplatin-resistant A549 cells.

d Resistance index: IC_50(A549/DOX)_/IC_50(A549)_.

e Resistance index: IC_50(A549/DDP)_/IC_50(A549)_.

f Dihydroartemisinin.

The cytotoxic results showed that all the desired hybrids (IC_50_: >100 *μ*M) were non-cytotoxic towards normal lung epithelial cell line (BEAS-2B), and nine of them (**5c**, **6a**, **6c**, **6e-j**) had the selectivity index (SI: IC_50(BEAS-2B)_/IC_50(A549)_) values > 1.0. In addition, the desired hybrids possessed the activity in the same level against both drug-sensitive and multidrug-resistant A549 lung cancer cell lines, and the drug resistance index (RI: IC_50(MDR__A549)_/IC_50(A549)_) values were 0.54–1.68, demonstrating their potential to overcome drug resistance.

In particular, hybrids **6a, e** (IC_50_: 5.72–9.84 *μ*M) showed pronounced activity against A549, A549/DOX and A549/DDP lung cancer cell lines. The activity of hybrids **6a, e** was not inferior to that of cisplatin (IC_50_: 9.38 *μ*M) and doxorubicin (IC_50_: 4.06 *μ*M) against A549 cells. These two hybrids were 1.5–10.8 times superior to cisplatin (IC_50_: 19.74 and 66.89 *μ*M) and doxorubicin (IC_50_: 54.32 and 15.10 *μ*M) against multidrug-resistant A549/DOX and A549/DDP lung cancer cell lines. Moreover, the SI index values of hybrids **6a, e** were >16.6, revealing that both of them possessed high specifity.

The metabolic stability of hybrids **6a, e** determined in mouse and human microsomes (Table 3) illustrated that both hybrids **6a, e** (liver microsomes: 51-70%) exhibited good microsomal stability, and hybrid **6a** (liver microsomes: 70 and 62%) was more stable than **6e** (liver microsomes: 54 and 51%) in both mouse and human microsomes.

**TABLE 3:** *In vitro* stability of hybrids **6a,e**.

Compd	Liver microsomes [%]	
	Mouse	Human
**6a**	70	62
**6e**	54	51

CD-1 mice model was administered with single intravenous 4) dose of 30 mg/kg to test the pharmacokinetic properties of hybrid **6a, e**. Hybrids **6a, e** showed the maximum plasma concentrations (*C*_max_) of 18.9 and 10.7 *μ*M, area under curve (AUC) of 2,352 and 1,670 ng^**.**^h/ml, half-lives (t_1/2_) of 3.1 and 4.7 h, peak time of 16 and 12 min, clearance rates (Cl) of 3.74 and 2.69 L/h/kg, and bioavailability of 39.7 and 30.8% (Table 4).

**TABLE 4:** Pharmacokinetic properties of hybrids **6a,e** in mice.

Parameter	Compd	
	6a	6e
*C*_max_ (*μ*M)	18.9	10.7
AUC (ng^**.**^h/ml)	2,352	1,670
t_1/2_ (h)	3.1	4.7
t_max_ (min)	16	12
Cl (L/h/kg)	3.74	2.69
*F* (%)	39.7	30.8

## Conclusion

In this study, we designed and synthesized fifteen novel dihydroartemisinin-isatin hybrids **5a-e** and **6a-j** with three-carbon linker. Their antiproliferative activity against both drug-sensitive and multidrug-resistant lung cancer cell lines was also evaluated. Among them, hybrids **6a, e** (IC_50_: 5.72–9.84 *μ*M) were highly potent against the three tested lung cancer cell lines, and the activity was comparable or superior to that of cisplatin (IC_50_: 9.38–66.89 *μ*M) and doxorubicin (IC_50_: 4.06–54.32 *μ*M). These two hybrids (IC_50_: >100 *μ*M) also demonstrated non-cytotoxicity towards normal lung epithelial cell line (BEAS-2B), and the SI index values of these two hybrids were >16.6, demonstrating their excellent safety profile. The RI values of hybrids **6a, e** were 1.03–1.66, indicating that these two hybrids could overcome drug resistance. In addition, hybrid **6a** also exhibited good stability in mouse and human microsomes and excellent pharmacokinetic properties. From these results, hybrid **6a** could be considered as a promising anti-lung cancer chemotherapeutic candidate for further evaluations.

## Data Availability

The original contributions presented in the study are included in the article/Supplementary Materials, further inquiries can be directed to the corresponding authors.
