# Supplementary material for: Three-Carbon Linked Dihydroartemisinin-Isatin Hybrids: Design, Synthesis and Their Antiproliferative Anticancer Activity
Source: Front Pharmacol. 2022 Jan 26;13:834317. doi: 10.3389/fphar.2022.834317 (PMC8826081; doi:10.3389/fphar.2022.834317)
Supplement: Supplementary file 1 [file DataSheet1.docx]

**Three-carbon linked dihydroartemisinin-isatin hybrids: Design, Synthesis and their antiproliferative anticancer activity**

Min Dong^1,#^, Guili Zheng^1,#^, Feng Gao^2,*^, Min Li^3,*^, Chen Zhong^1,*^

^1^ Department of Oncology, No. 960 Hospital of PLA, Jinan, China

^2^ Key Laboratory for Experimental Teratology of the Ministry of Education and Center for Experimental Nuclear Medicine, School of Basic Medical Sciences, Cheeloo College of Medicine, Shandong University, Jinan, China

^3^ Department of Nuclear Medicine, No. 960 Hospital of PLA, Jinan, China

^#^ Min Dong and Guili Zheng contribute equally.

**Experimental section**

1. **Materials**

^1^H NMR and ^13^C NMR spectra were determined on a Varian Mercury-400 spectrometer in DMSO-*d*_6_, CDCl_3_ or CD_3_OD using tetramethylsilane (TMS) as an internal standard. Electrospray ionization (ESI) mass spectra were obtained on a MDSSCIEXQ-Tap mass spectrometer. Unless otherwise noted, the reagents were obtained from commercial supplier and were used without further purification. A549, A549/DOX, and A549/DDP lung cancer cell lines were purchased from the American Type Culture Collection (ATCC) and preserved by Center for Experimental Nuclear Medicine of Shandong University.

1. **Synthesis**

To a mixture of (5-substituted) isatins **1** (100 mmol) in DMF (100 mL), potassium carbonate (K_2_CO_3_, 200 mmol) was added. The mixture was stirred at room temperature for 1 h, and then 3-bromopropanol **2** (150 mmol) was added. The mixture was stirred overnight at room temperature, and then filtered. The filtrate was concentrated under reduced pressure and the residue was purified by silica gel chromatography eluted with PE to PE : EA = 2 : 1 to provide intermediates **3**.

To the mixture of intermediates **3** (120 mmol) and dihydroartemisinin **4** (100 mmol) in DCM (500 mL) was added boron trifluoride diethyl etherate (BF_3_**^.^**OEt_2_, 20 mL) at 0 ^o^C, and the mixture was stirred at room temperatire overnight. Sat. Na_2_CO_3_ (500 mL) was added to the mixture, and then the organic layer was separated. The organic layer was washed with H_2_O (500 mL) and brine (500 mL) in sequence, dried over anhydrous Na_2_SO_4_, filtered, and concentrated under reduced. The residue was purified by silica gel chromatography eluted with PE to PE : EA = 1 : 1 to give dihydroartemisinin-isatin hybrids **5a-e**.

To a solution of hybrids **5a-e** (1 mmol) and hydroxylamine/semicarbazide/thiosemicarbazide hydrochlorides (1.5 mmol) in a mixture of THF (10 mL) and H_2_O (10 mL), NaHCO_3_ (2 mmol) was added. The mixture was stirred at 60 ^o^C for 12 h, and then cooled to room temperature. The mixture was extracted with DCM (20 mL × 3). The combined organic layers were washed with H_2_O (30 mL) and brine (30 mL) in sequence, dried over anhydrous Na_2_SO_4_, filtered, and concentrated under reduced pressure. The residue was purified by silica gel chromatography eluted with PE to PE : EA = 1 : 1 to give dihydroartemisinin-isatin hybrids **6a-j**.

1. **Characterization**

1-(3-(((3*R*,5a*S*,6*R*,8a*S*,9*R*,12*R*,12a*R*)-3,6,9-trimethyldecahydro-12*H*-3,12-epoxy[1,2]dioxepino[4,3-i]isochromen-10-yl)oxy)propyl)indoline-2,3-dione (**5a**)

Brown solid. ^1^H NMR (600 Hz, DMSO-*d*_6_) δ 0.92-0.99 (m, 7H), 1.25-1.39 (m, 3H), 1.41-1.49 (m, 4H), 1.66-1.69 (m, 1H), 1.63-1.74 (m, 3H), 1.78-2.06 (m, 4H), 2.35-2.67 (m, 2H), 3.46-3.51 (m, 1H), 3.69-4.07 (m, 3H), 4.81 (d, *J* = 2.0 Hz, 1H), 5.42 (s, 1H), 6.92-7.13 (m, 2H), 7.55-7.63 (m, 2H). ^13^C NMR (150 Hz, DMSO-*d*_6_) 183.45, 158.13, 150.97, 138.62, 138.25, 125.53, 125.30, 123.70, 123.60, 117.66, 110.70, 110.00, 104.31, 104.18, 102.15, 100.33, 81.03, 80.41, 66.05, 65.50, 58.89, 52.55, 51.66, 45.37, 44.37, 37.74, 37.45, 37.38, 36.40, 34.63, 30.86, 27.74, 26.17, 26.06, 24.67, 24.59, 20.37, 13.09, 12.72. HRMS-ESI: m/z Calcd for C_26_H_33_NO_7_Na [M+Na]^+^: 494.2149; Found: 494.2144.


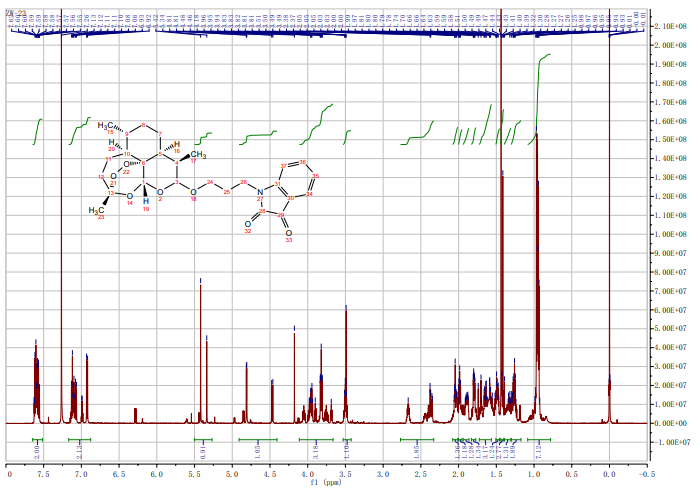


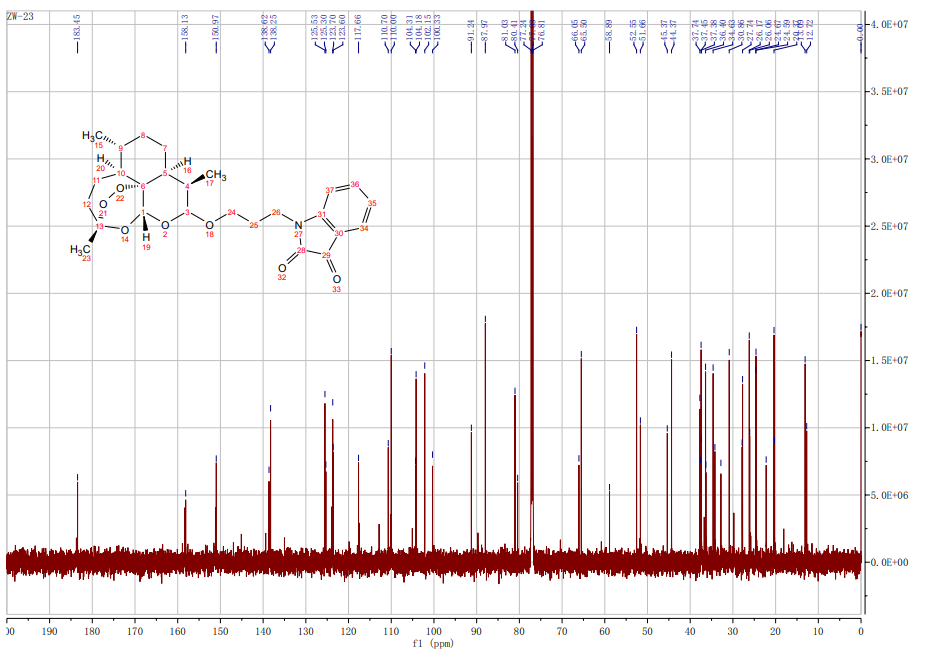


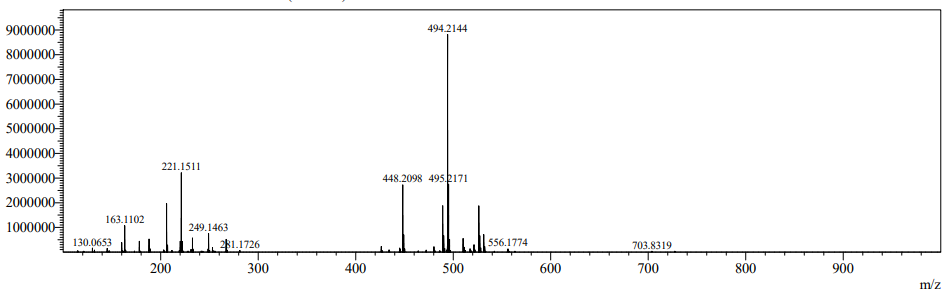


5-fluoro-1-(3-(((3*R*,5a*S*,6*R*,8a*S*,9*R*,12*R*,12a*R*)-3,6,9-trimethyldecahydro-12*H*-3,12-epoxy[1,2]dioxepino[4,3-i]isochromen-10-yl)oxy)propyl)indoline-2,3-dione (**5b**)

Brown solid. ^1^H NMR (600 Hz, DMSO-*d*_6_) δ 0.92-1.12 (m, 7H), 1.23-1.28 (m, 1H), 1.33-1.39 (m, 1H), 1.43-1.59 (m, 4H), 1.63-1.66 (m, 1H), 1.67-2.08 (m, 7H), 2.35-2.40 (m, 1H), 2.66-2.68 (m, 1H), 3.48-3.51 (m, 1H), 3.81-3.96 (m, 3H), 4.80 (d, *J* = 4.0 Hz, 1H), 5.42 (s, 1H), 6.90 (dd, *J* = 4.0, 8.0 Hz, 1H), 7.28-7.33 (m, 2H). 13C NMR (150 Hz, DMSO-d6) 182.85, 160.10, 158.47, 157.89, 146.99, 124.80, 124.63, 124.47, 118.28, 112.67, 112.51, 111.18, 111.12, 104.20, 102.18, 87.97, 81.00, 65.48, 58.91, 52.53, 44.33, 37.87, 37.46, 36.39, 30.84, 29.70, 27.64, 26.16, 24.66, 24.58, 20.36, 13.09. HRMS-ESI: m/z Calcd for C_26_H_32_FNO_7_Na [M+Na]^+^: 512.2055; Found: 512.2050.


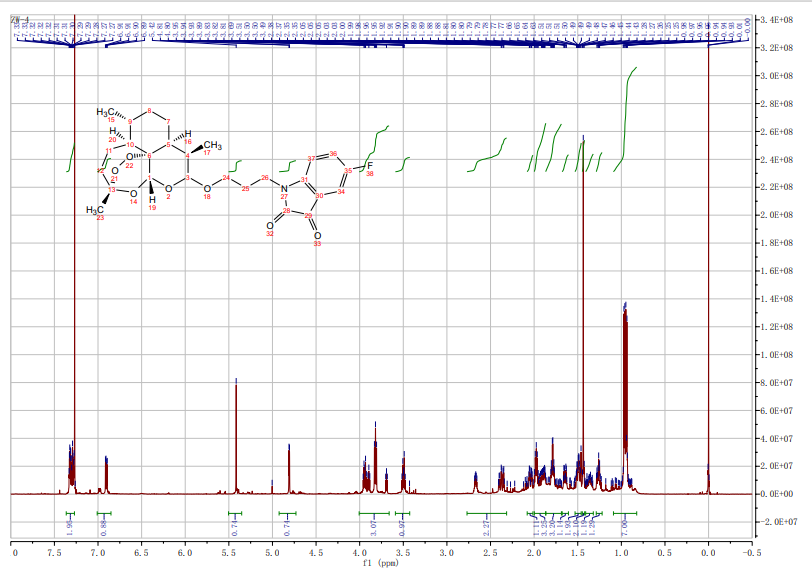


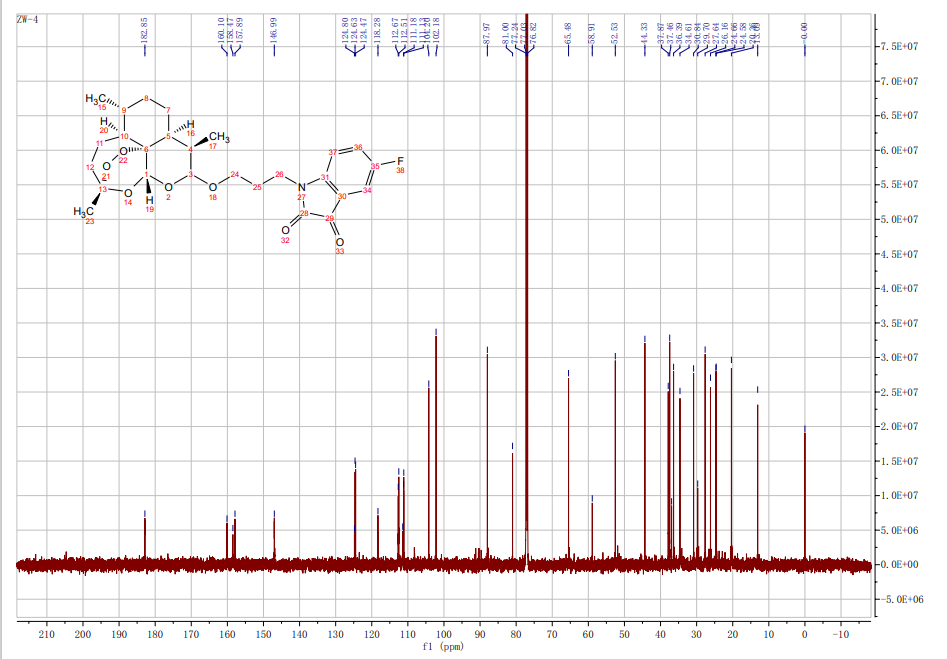


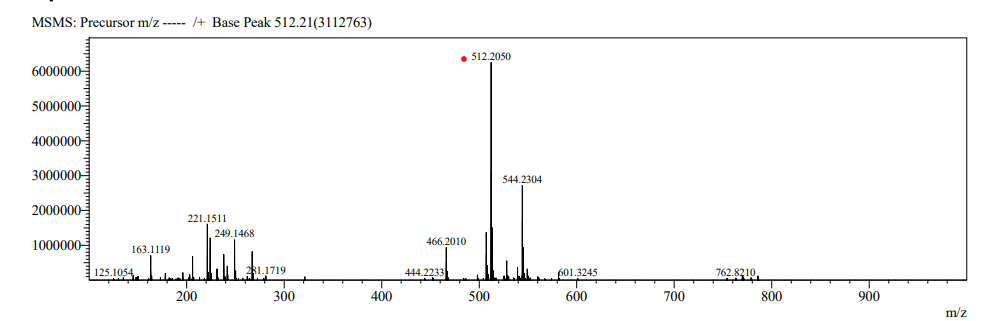


5-chloro-1-(3-(((3*R*,5a*S*,6*R*,8a*S*,9*R*,12*R*,12a*R*)-3,6,9-trimethyldecahydro-12*H*-3,12-epoxy[1,2]dioxepino[4,3-i]isochromen-10-yl)oxy)propyl)indoline-2,3-dione (**5c**)

Brown solid. ^1^H NMR (600 Hz, DMSO-*d*_6_) δ 0.84-0.99 (m, 7H), 1.25-1.33 (m, 3H), 1.42-1.58 (m, 5H), 1.64-1.70 (m, 1H), 1.77-1.82 (m, 1H), 1.87-2.06 (m, 4H), 2.35-2.41 (m, 1H), 2.66-2.68 (m, 1H), 3.46-3.51 (m, 1H), 3.67-4.14 (m, 3H), 4.81 (d, *J* = 2.0 Hz, 1H), 5.46 (s, 1H), 6.82 (d, *J* = 4.0 Hz, 1H), 7.32 (dd, *J* = 2.0, 4.0 Hz, 1H), 8.07 (d, *J* = 2.0 Hz, 1H). ^13^C NMR (150 Hz, DMSO-*d*_6_) 163.80, 143.57, 141.96, 132.17, 131.79, 128.45, 128.15, 127.92, 116.66, 110.31, 109.53, 104.37, 104.24, 102.18, 100.35, 91.24, 87.99, 81.08, 80.41, 66.11, 65.59, 60.46, 58.74, 52.55, 51.65, 45.35, 44.29, 37.43, 37.37, 36.40, 36.32, 34.64, 34.22, 30.87, 27.93, 26.15, 26.03, 24.66, 24.58, 20.38, 20.28, 12.09, 12.72. HRMS-ESI: m/z Calcd for C_26_H_32_ClNO_7_Na [M+Na]^+^: 528.1760; Found: 528.1746.


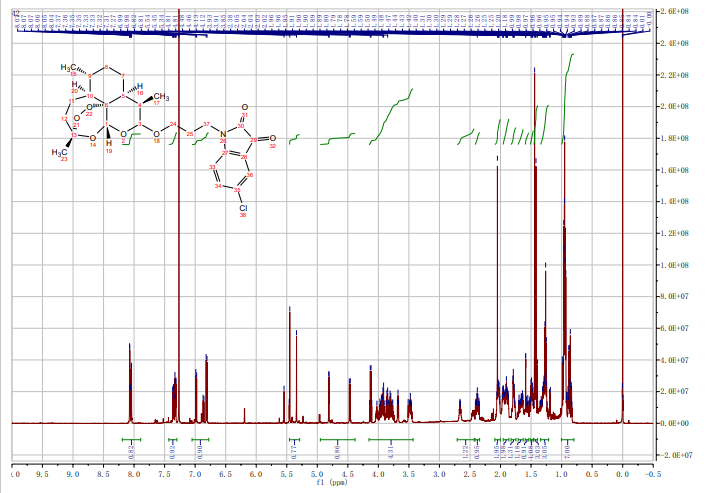


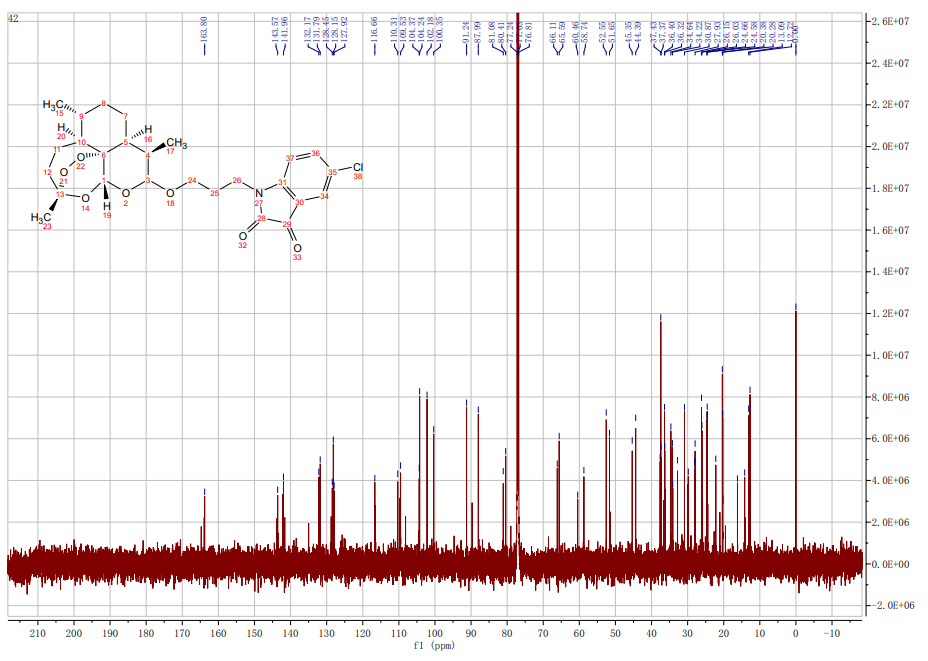


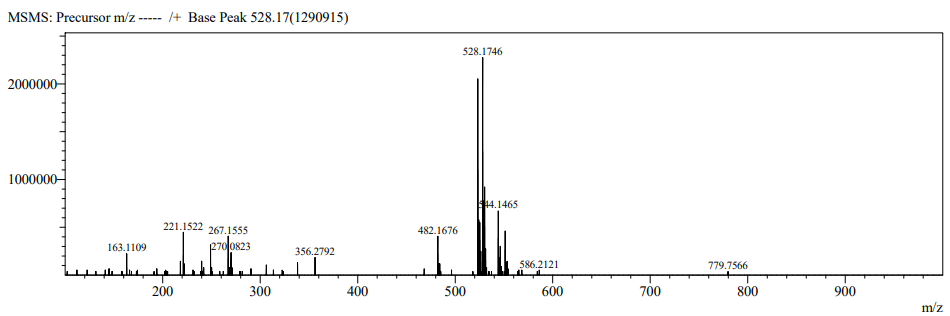


5-methyl-1-(3-(((3*R*,5a*S*,6*R*,8a*S*,9*R*,12*R*,12a*R*)-3,6,9-trimethyldecahydro-12*H*-3,12-epoxy[1,2]dioxepino[4,3-i]isochromen-10-yl)oxy)propyl)indoline-2,3-dione (**5d**)

Brown soild. ^1^H NMR (600 Hz, DMSO-*d*_6_) δ 0.92-1.02 (m, 7H), 1.25-1.37 (m, 3H), 1.41-1.59 (m, 4H), 1.63-1.70 (m, 1H), 1.77-1.82 (m, 1H), 1.87-1.99 (m, 3H), 2.02-2.06 (m, 2H), 2.34 (s, 3H, CH3), 2.36-2.44 (m, 1H), 2.66-2.68 (m, 1H), 3.46-3.51 (m, 1H), 3.66-4.14 (m, 3H), 4.80 (d, *J* = 2.0 Hz, 1H), 5.54 (s, 1H), 6.82 (d, J = 4.0 Hz, 1H), 7.36-7.42 (m, 2H). ^13^C NMR (150 Hz, DMSO-*d*_6_) 183.98, 183.74, 158.48, 158.25, 148.79, 138.99, 138.80, 138.63, 133.53, 133.38, 125.92, 125.85, 125.63, 117.68, 117.57, 110.49, 110.00, 109.82, 104.29, 104.16, 102.14, 100.32, 91.23, 87.95, 81.03, 80.41, 66.06, 65.51, 58.86, 52.55, 51.66, 45.37, 44.38, 37.44, 36.40, 27.75, 26.16, 24.66, 24.57, 20.66, 20.65, 20.37, 14.20, 13.09, 12.72. HRMS-ESI: m/z Calcd for C_27_H_35_NO_7_Na [M+Na]^+^: 508.2306; Found: 508.2277.


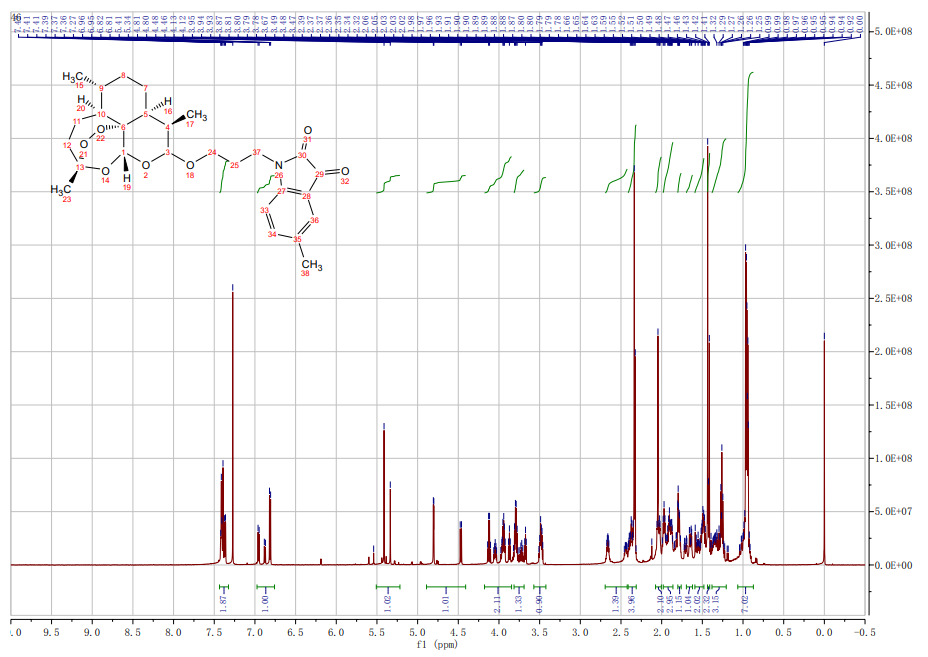


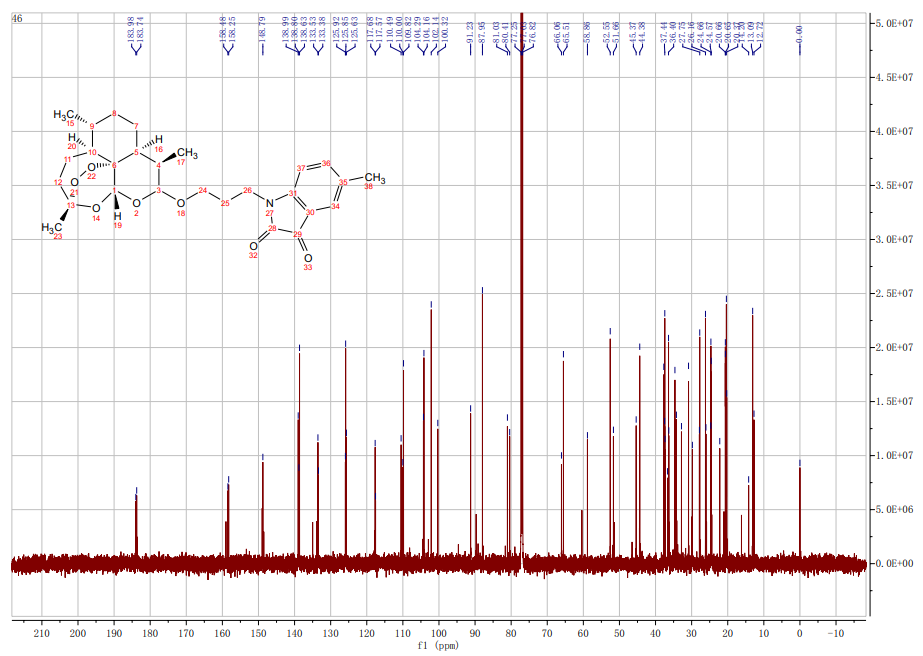


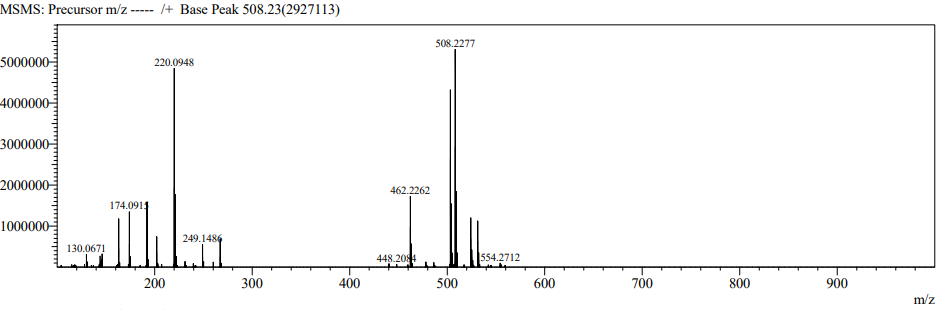


5-methoxy-1-(3-(((3*R*,5a*S*,6*R*,8a*S*,9*R*,12*R*,12a*R*)-3,6,9-trimethyldecahydro-12*H*-3,12-epoxy[1,2]dioxepino[4,3-i]isochromen-10-yl)oxy)propyl)indoline-2,3-dione (**5e**)

Brown solid. ^1^H NMR (600 Hz, CD_3_OD) 0.93-0.99 (m, 7H), 1.20-1.39 (m, 5H), 1.41-1.53 (m, 2H), 1.61-1.84 (m, 2H), 1.89-2.07 (m, 5H), 2.29-2.56 (m, 2H), 3.40-3.59 (m, 1H), 3.79-4.13 (m, 6H), 4.55-4.75 (m, 1H), 5.44 (s, 1H), 6.93-7.25 (m, 3H). ^13^C NMR (150 Hz, CD_3_OD) 187.57, 162.80, 160.63, 148.50, 127.70, 127.53, 122.29, 115.64, 115.32, 113.21, 113.09, 107.99, 106.12, 104.20, 95.18, 91.82, 84.75, 84.24, 70.05, 69.68, 56.53, 41.42, 41.01, 39.95, 39.93, 34.88, 31.31, 28.61, 28.35, 28.22, 23.29, 15.90, 15.58. HRMS-ESI: m/z Calcd for C_27_H_35_NO_8_Na [M+Na]^+^: 524.2255; Found: 524.2227.


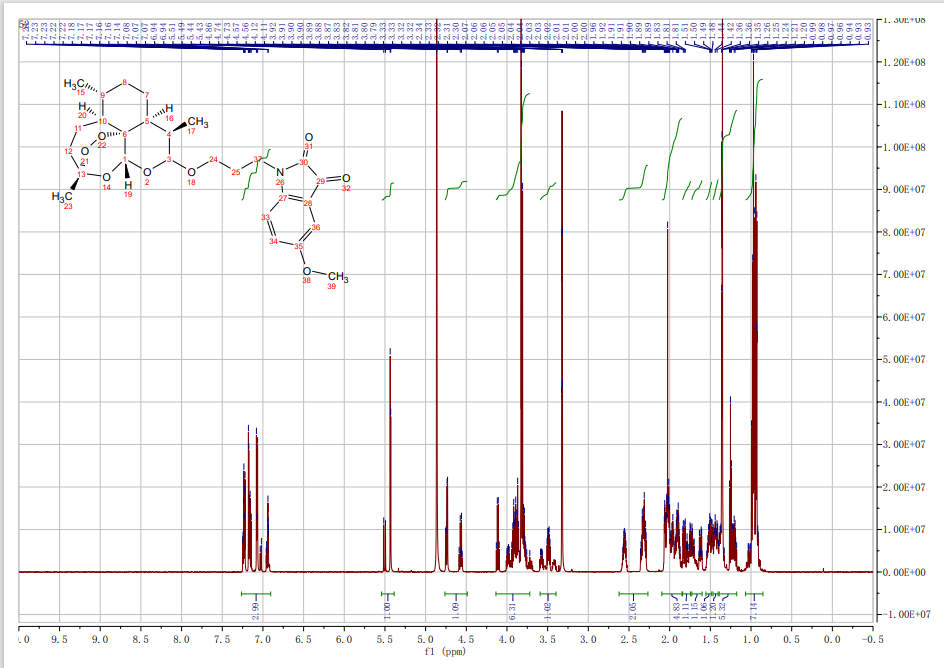


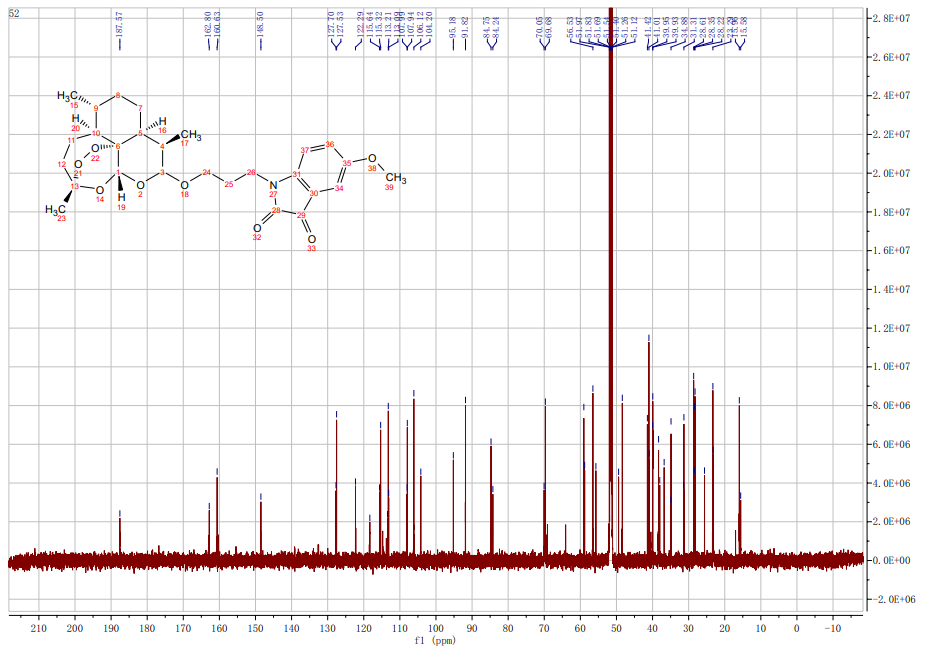


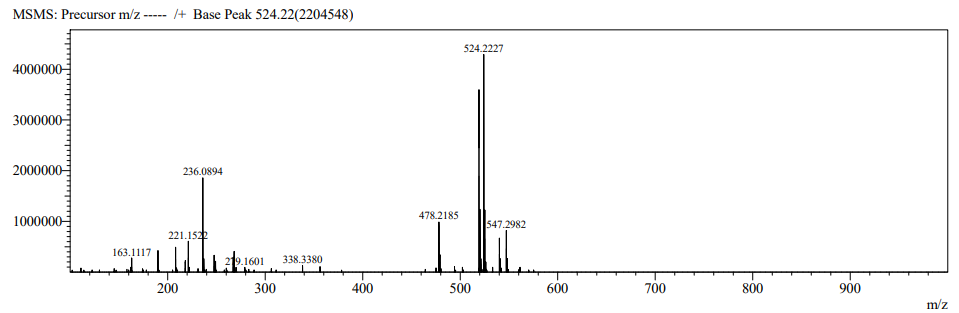


3-(hydroxyimino)-1-(3-(((3*R*,5a*S*,6*R*,8a*S*,9*R*,12*R*,12a*R*)-3,6,9-trimethyldecahydro-12*H*-3,12-epoxy[1,2]dioxepino[4,3-i]isochromen-10-yl)oxy)propyl)indolin-2-one (**6a**)

Yellow solid. ^1^H NMR (600 Hz, DMSO-*d*_6_) δ 0.86-0.99 (m, 7H), 1.26-1.57 (m, 7H), 1.64-1.90 (m, 3H), 1.95-2.06 (m, 3H), 2.25-2.66 (m, 3H), 3.45-3.52 (m, 1H), 3.78-4.12 (m, 3H), 4.95 (d, *J* = 2.0 Hz, 1H), 5.47 (s, 1H), 6.88 (d, *J* = 4.0 Hz, 1H), 7.09 (t, *J* = 4.0 Hz, 1H), 7.36 (t, *J* = 4.0 Hz, 1H), 8.12 (d, *J* = 4.0 Hz, 1H), 11.04 (brs, 1H, NOH). ^13^C NMR (150 Hz, DMSO-*d*_6_) 164.31, 164.22, 144.55, 144.34, 143.75, 143.62, 132.47, 132.17, 128.32, 128.14, 123.14, 123.06, 115.80, 115.65, 109.09, 108.53, 104.33, 104.21, 102.15, 100.31, 91.24, 87.98, 81.12, 80.43, 66.30, 65.65, 60.47, 52.57, 51.66, 45.39, 44.43, 37.46, 37.41, 36.42, 30.90, 28.00, 26.16, 24.66, 24.58, 20.38, 14.20, 13.09, 12.73. HRMS-ESI: m/z Calcd for C_26_H_34_N_2_O_7_Na [M+Na]^+^: 509.2258; Found: 509.2241.


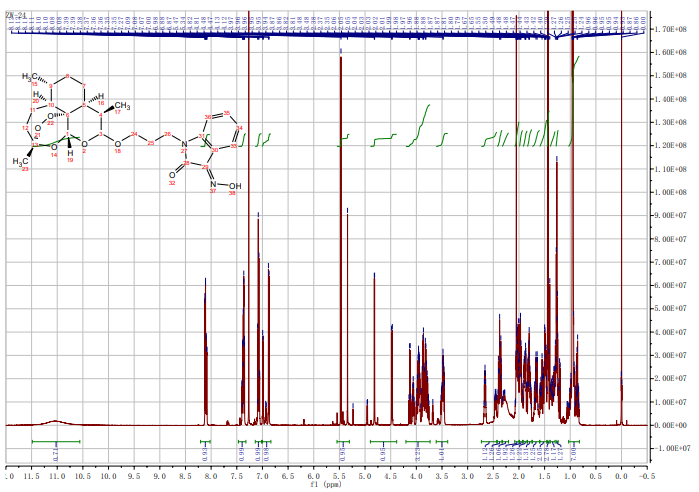


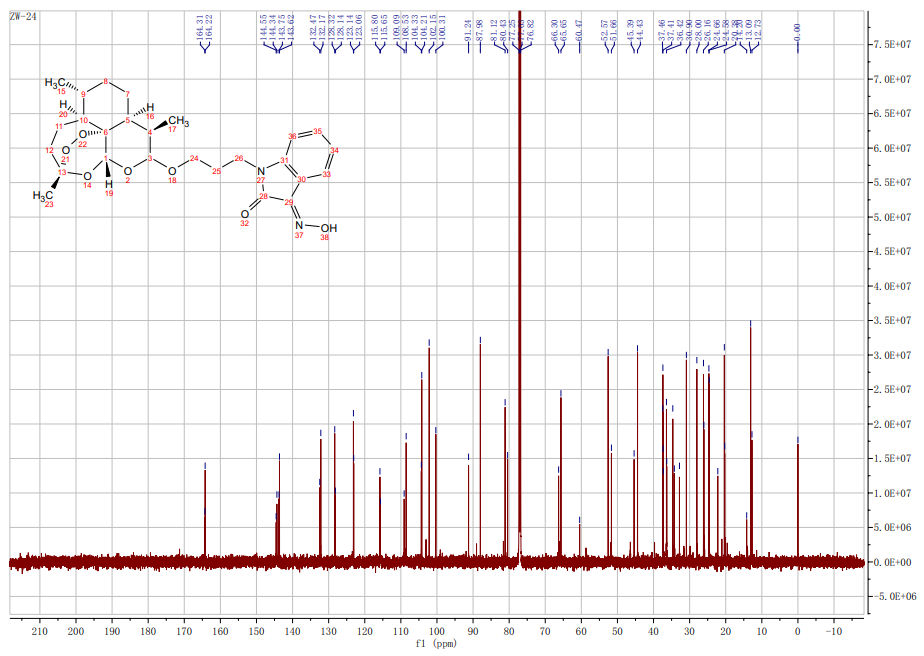


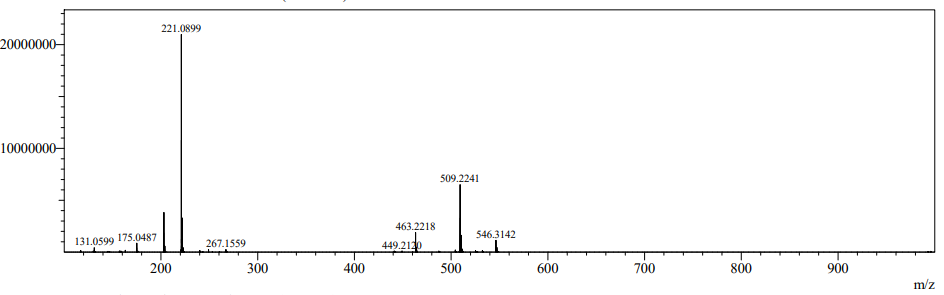


2-(2-oxo-1-(3-(((3*R*,5a*S*,6*R*,8a*S*,9*R*,12*R*,12a*R*)-3,6,9-trimethyldecahydro-12*H*-3,12-epoxy[1,2]dioxepino[4,3-i]isochromen-10-yl)oxy)propyl)indolin-3-ylidene)hydrazine-1-carboxamide (**6b**)

Yellow solid. ^1^H NMR (600 Hz, DMSO-*d*_6_) δ 0.91-1.06 (m, 7H), 1.24-1.37 (m, 2H), 1.40-1.58 (m, 4H), 1.63-1.83 (m, 4H), 1.87-2.06 (m, 3H), 2.35-2.68 (m, 2H), 3.43-3.53 (m, 1H), 3.68-4.06 (m, 3H), 4.81 (d, *J* = 2.0 Hz, 1H), 5.42 (s, 1H), 6.82 (d, *J* = 4.0 Hz, 1H), 7.12 (t, *J* = 4.0 Hz, 1H), 7.56-7.62 (m, 2H). ^13^C NMR (150 Hz, DMSO-*d*_6_) 183.45, 158.14, 150.97, 138.63, 138.26, 125.52, 125.30, 123.70, 123.60, 117.65, 110.70, 110.00, 104.31, 104.18, 102.15, 100.34, 91.24, 87.96, 81.03, 80.41, 66.06, 65.50, 52.55, 51.66, 45.36, 44.37, 37.74, 37.45, 37.37, 36.40, 34.63, 30.86, 27.73, 26.16, 24.66, 24.58, 20.37, 20.28, 13.09, 12.72.


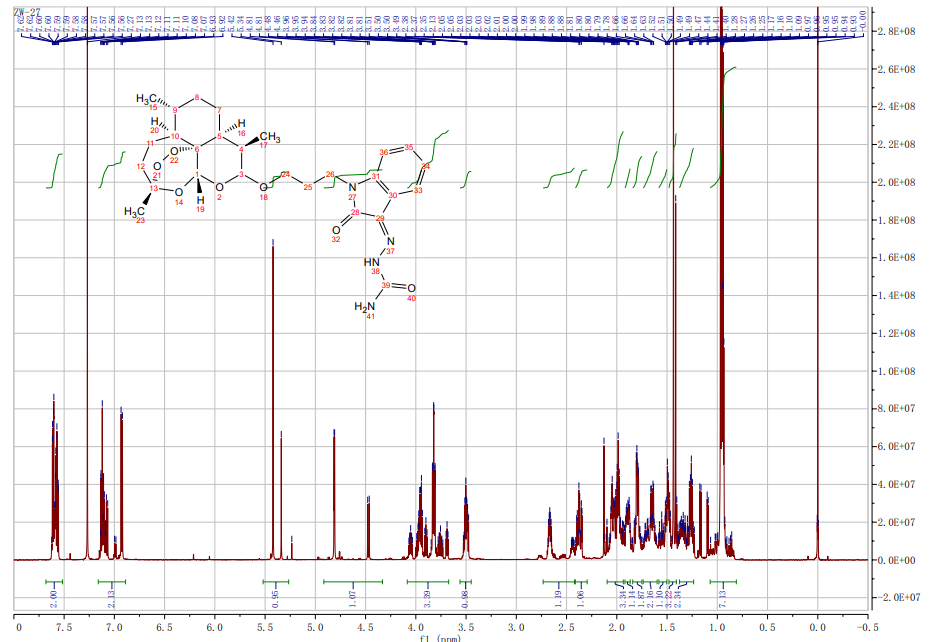


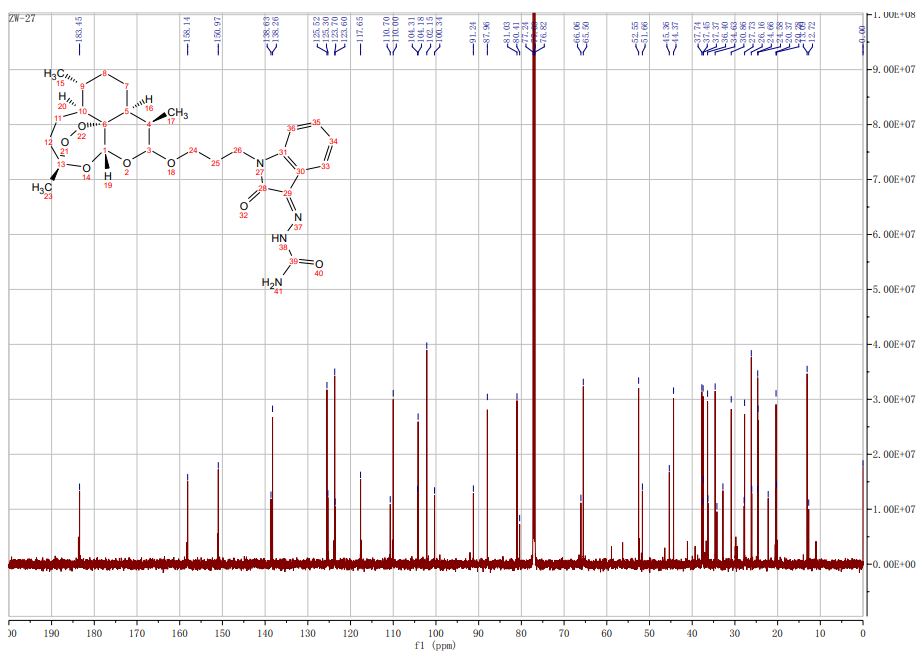


2-(2-oxo-1-(3-(((3*R*,5a*S*,6*R*,8a*S*,9*R*,12*R*,12a*R*)-3,6,9-trimethyldecahydro-12*H*-3,12-epoxy[1,2]dioxepino[4,3-i]isochromen-10-yl)oxy)propyl)indolin-3-ylidene)hydrazine-1-carbothioamide (**6c**)

Yellow solid. ^1^H NMR (600 Hz, DMSO-*d*_6_) δ 0.86-0.99 (m, 7H), 1.23-1.34 (m, 2H), 1.41-1.59 (m, 5H), 1.65-1.71 (m, 1H), 1.78-2.07 (m, 6H), 2.34-2.68 (m, 2H), 3.44-3.51 (m, 1H), 3.77-4.13 (m, 3H), 4.80 (d, *J* = 4.0 Hz, 1H), 5.48 (s, 1H), 6.58 (brs, 1H, CSNH), 6.92 (d, *J* = 4.0 Hz, 1H), 7.12 (t, *J* = 4.0 Hz, 1H), 7.38 (t, *J* = 4.0 Hz, 1H), 7.53 (brs, 1H, CSNH), 7.58 (d, *J* = 4.0 Hz, 1H), 12.88 (brs, NNHCS). ^13^C NMR (150 Hz, DMSO-*d*_6_) 180.02, 161.05, 143.27, 132.03, 131.81, 131.51, 123.22, 123.13, 120.99, 120.82, 119.47, 109.86, 109.33, 104.31, 104.18, 102.17, 100.30, 91.25, 87.96, 81.11, 80.43, 66.16, 65.32, 60.41, 52.65, 51.67, 45.39, 44.41, 37.36, 36.42, 34.71, 30.89, 27.89, 26.18, 24.68, 24.55, 20.45, 14.20, 13.08, 12.70. HRMS-ESI: m/z Calcd for C_27_H_36_N_2_O_6_SNa [M+Na]^+^: 567.2248; Found: 567.2243.


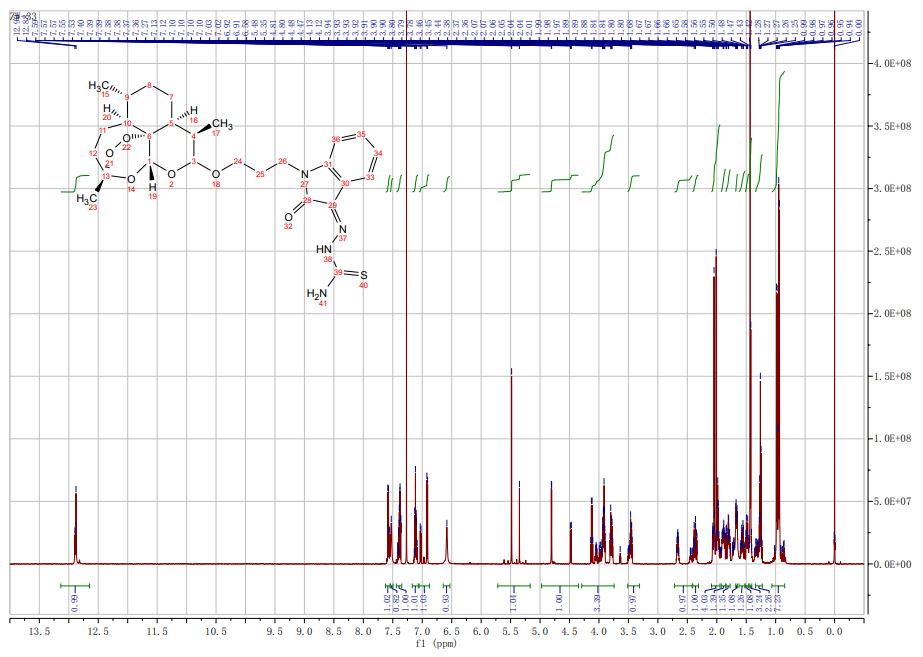


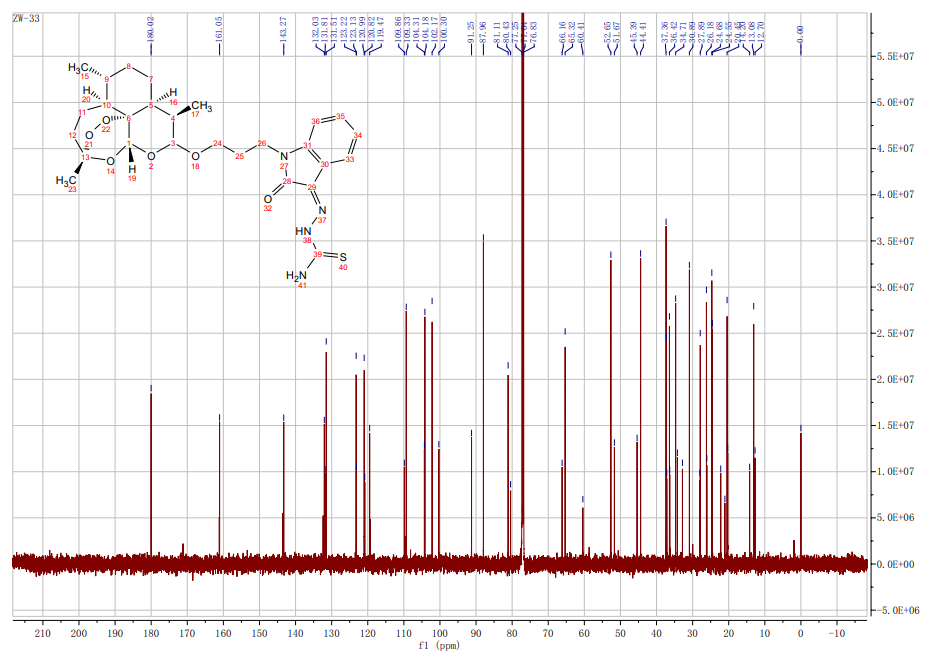


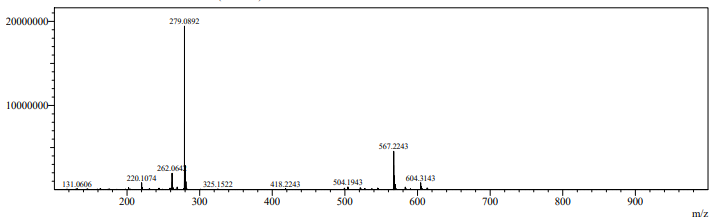


2-(5-fluoro-2-oxo-1-(3-(((3*R*,5a*S*,6*R*,8a*S*,9*R*,12*R*,12a*R*)-3,6,9-trimethyldecahydro-12*H*-3,12-epoxy[1,2]dioxepino[4,3-i]isochromen-10-yl)oxy)propyl)indolin-3-ylidene)hydrazine-1-carboxamide (**6d**)

Yellow solid. ^1^H NMR (600 Hz, DMSO-*d*_6_) δ 0.92-1.02 (m, 7H), 1.17-1.38 (m, 3H), 1.39-1.44 (m, 3H), 1.47-1.72 (m, 3H), 1.77-1.80 (m, 1H), 1.88-2.06 (m, 4H), 2.35-2.68 (m, 2H), 3.48-3.54 (m, 1H), 3.75-4.03 (m, 3H), 4.80 (d, *J* = 4.0 Hz, 1H), 5.42 (s, 1H), 6.89-7.12 (m, 1H), 7.28-7.35 (m, 2H). ^13^C NMR (150 Hz, DMSO-*d*_6_)182.85, 160.10, 158.45, 158.19, 157.89, 147.30, 146.99, 125.11, 124.95, 124.63, 124.47, 118.24, 118.04, 112.28, 112.16, 111.13, 104.34, 104.20, 102.89, 102.19, 100.37, 91.23, 87.97, 81.00, 80.39, 65.96, 65.48, 52.53, 51.64, 45.33, 44.34, 37.46, 37.38, 36.39, 32.81, 30.84, 27.93, 26.03, 24.69, 24.58, 20.27, 13.09, 12.70.


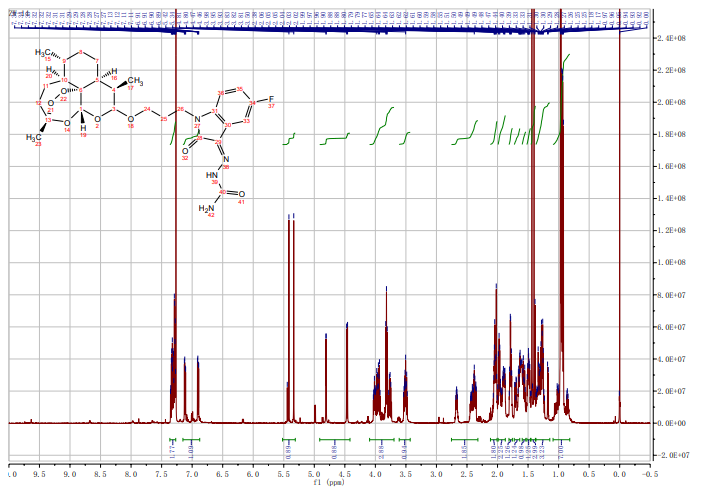


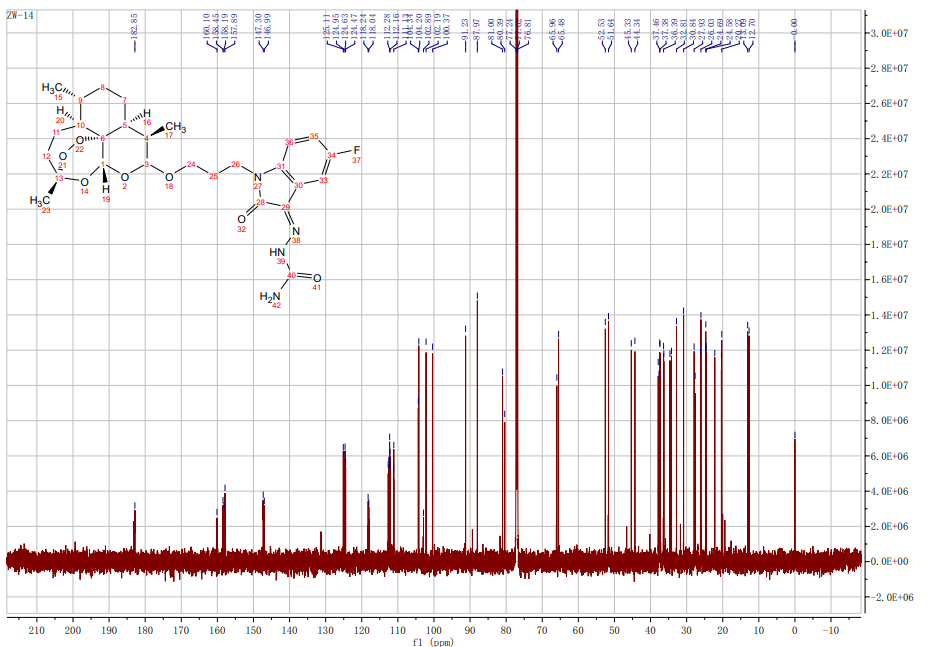


2-(5-fluoro-2-oxo-1-(3-(((3*R*,5a*S*,6*R*,8a*S*,9*R*,12*R*,12a*R*)-3,6,9-trimethyldecahydro-12*H*-3,12-epoxy[1,2]dioxepino[4,3-i]isochromen-10-yl)oxy)propyl)indolin-3-ylidene)hydrazine-1-carbothioamide (**6e**)

Yellow solid. ^1^H NMR (600 Hz, DMSO-*d*_6_) δ 0.86-1.10 (m, 7H), 1.25-1.35 (m, 2H), 1.41-1.59 (m, 5H), 1.65-1.84 (m, 3H), 1.88-2.11 (m, 4H), 2.34-2.66 (m, 2H), 3.44-3.50 (m, 1H), 3.76-4.02 (m, 3H), 4.80 (d, *J* = 4.0 Hz, 1H), 5.48 (s, 1H), 6.61 (s, 1H, CSNH), 7.01-7.12 (m, 2H), 7.30 (dd, *J* = 2.0, 4.0 Hz, 1H), 7.51 (s, 1H, CSNH), 12.88 (s, 1H, NNHCS). ^13^C NMR (150 Hz, DMSO-*d*_6_) 180.02, 161.12, 161.03, 158.56, 139.51, 139.20, 131.28, 120.77, 120.72, 118.24, 118.07, 117.85, 117.69, 110.15, 110.10, 108.58, 108.41, 104.33, 104.20, 100.34, 91.24, 87.97, 81.09, 80.42, 66.08, 65.28, 52.64, 51.66, 45.36, 44.38, 37.36, 36.41, 34.70, 30.88, 27.82, 26.17, 24.69, 24.55, 20.45, 20.28, 13.08, 12.69. HRMS-ESI: m/z Calcd for C_27_H_35_FN_4_O_6_SNa [M+Na]^+^: 585.2154; Found: 585.2131.


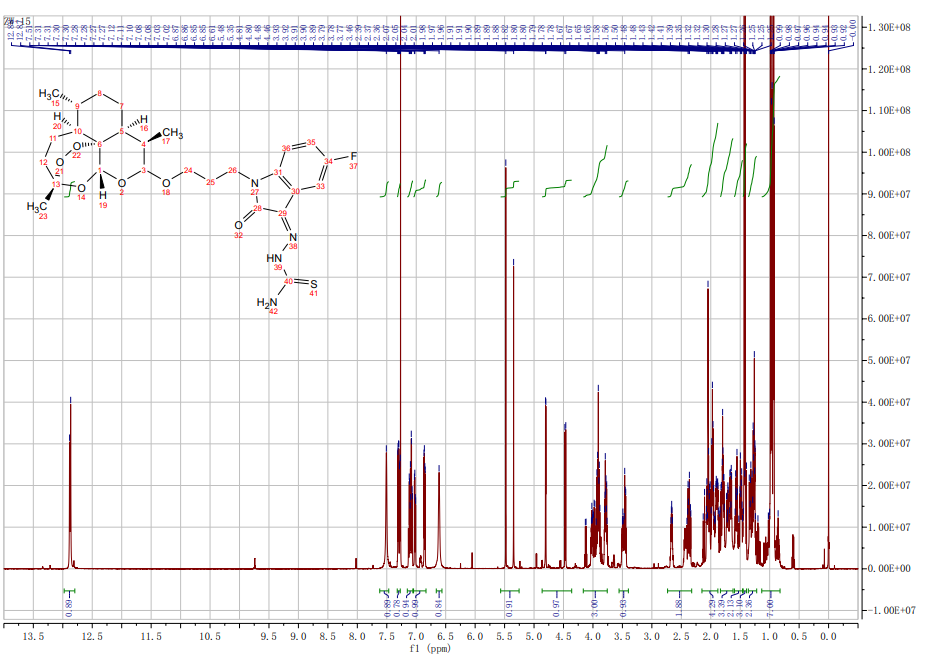


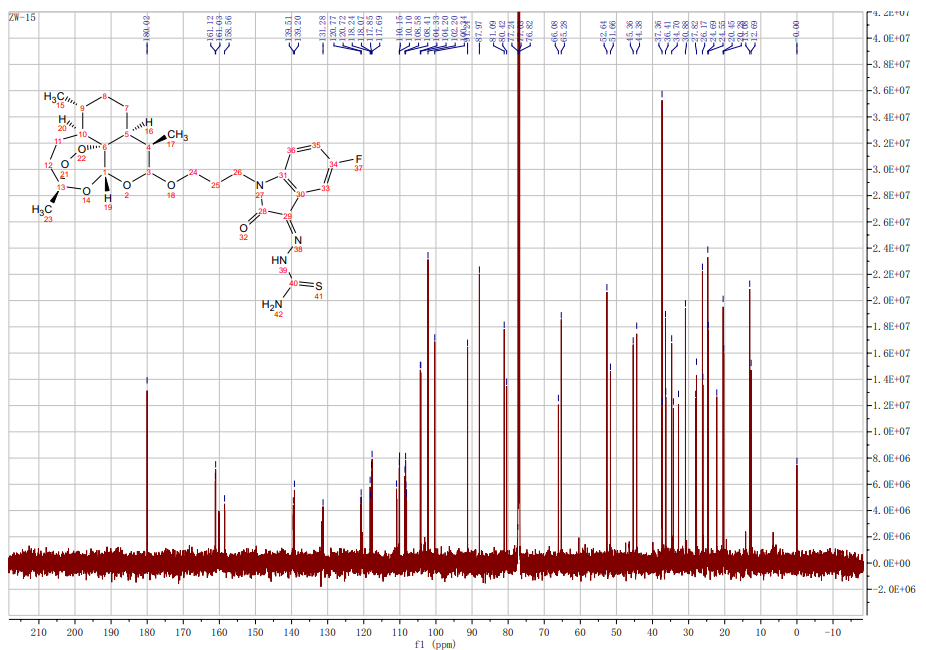


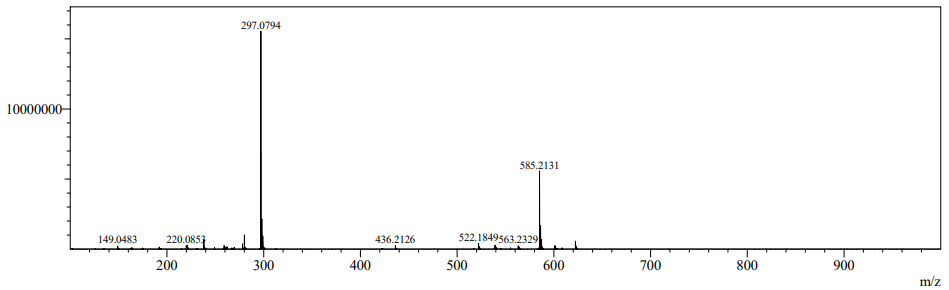


5-chloro-3-(hydroxyimino)-1-(3-(((3*R*,5a*S*,6*R*,8a*S*,9*R*,12*R*,12a*R*)-3,6,9-trimethyldecahydro-12*H*-3,12-epoxy[1,2]dioxepino[4,3-i]isochromen-10-yl)oxy)propyl)indolin-2-one (**6f**)

Yellow solid. ^1^H NMR (600 Hz, DMSO-*d*_6_) δ 0.94-1.02 (m, 5H), 1.08 (d, J = 8.0 Hz, 2H), 1.22-1.33 (m, 3H), 1.42-1.50 (m, 5H), 1.55-1.72 (m, 3H), 1.88-2.05 (m, 4H), 2.27-2.33 (m, 1H), 3.56-3.60 (m, 1H), 3.84-3.98 (m, 3H), 4.96 (d, *J* = 4.0 Hz, 1H), 5.46 (s, 1H), 6.88 (d, *J* = 4.0 Hz, 1H), 7.32 (dd, *J* = 2.0, 4.0 Hz, 1H), 8.06 (d, *J* = 2.0 Hz, 1H), 11.44 (brs, 1H, NOH). ^13^C NMR (150 Hz, DMSO-*d*_6_) 163.93, 143.63, 142.10, 134.99, 131.82, 128.35, 128.00, 116.60, 109.91, 103.15, 103.01, 89.71, 89.16, 81.63, 66.06, 58.81, 51.83, 46.48, 44.45, 39.90, 37.67, 37.49, 37.28, 36.48, 36.24, 34.41, 34.11, 31.66, 30.00, 29.80, 27.81, 25.92, 24.67, 24.42, 20.29, 20.07, 19.52, 16.19. HRMS-ESI: m/z Calcd for C_26_H_33_N_2_O_7_ClNa [M+Na]^+^: 543.1869; Found: 543.1846.


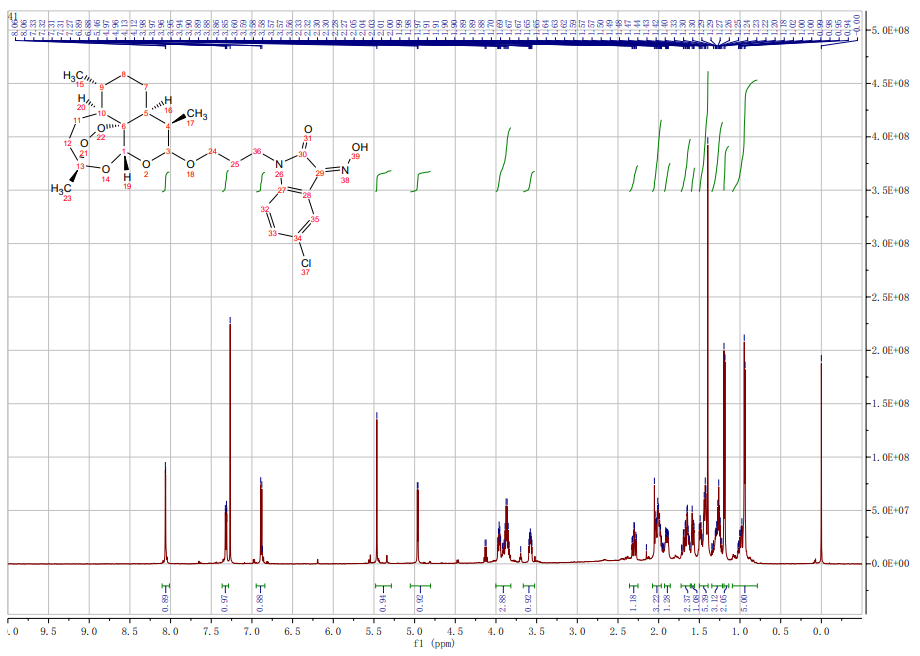


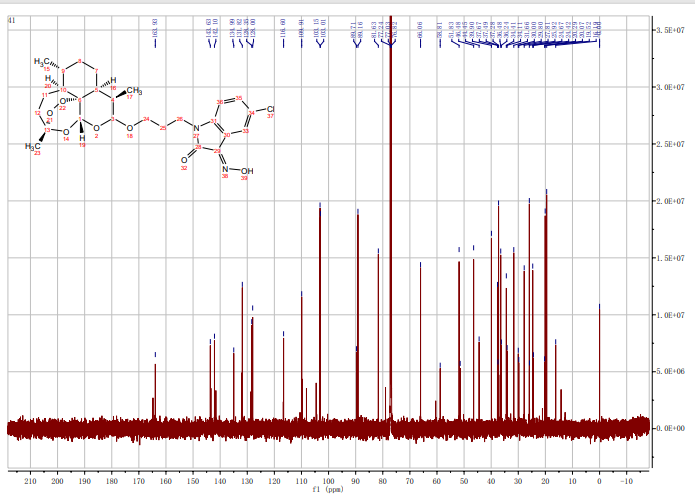


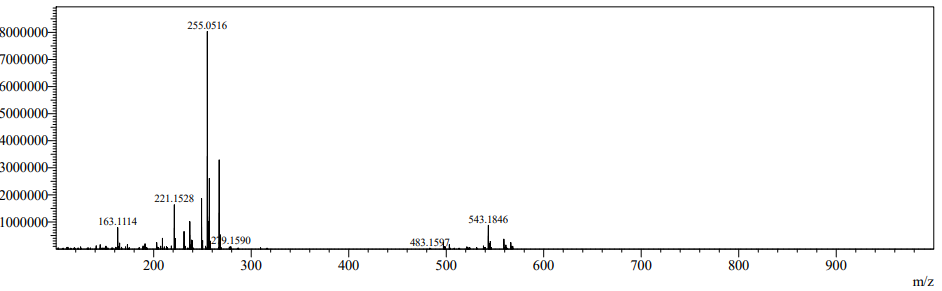


3-(hydroxyimino)-5-methyl-1-(3-(((3*R*,5a*S*,6*R*,8a*S*,9*R*,12*R*,12a*R*)-3,6,9-trimethyldecahydro-12*H*-3,12-epoxy[1,2]dioxepino[4,3-i]isochromen-10-yl)oxy)propyl)indolin-2-one (**6g**)

Yellow solid. ^1^H NMR (600 Hz, DMSO-*d*_6_) δ 0.84-0.99 (m, 7H), 1.24-1.37 (m, 3H), 1.43-1.59 (m, 5H), 1.64-1.68 (m, 1H), 1.78-1.82 (m, 1H), 1.87-1.98 (m, 2H), 2.01-2.06 (m, 2H), 2.34 (s, 3H, CH3), 2.36-2.44 (m, 1H), 2.64-2.68 (m, 1H), 3.43-3.52 (m, 1H), 3.70-4.14 (m, 3H), 4.81 (d, *J* = 2.0 Hz, 1H), 5.45 (s, 1H), 6.76 (d, *J* = 4.0 Hz, 1H), 7.16 (d, *J* = 4.0 Hz, 1H), 7.95 (s, 1H). ^13^C NMR (150 Hz, DMSO-*d*_6_) 164.25, 144.55, 141.38, 134.99, 132.77, 132.63, 132.49, 128.96, 128.79, 115.78, 108.84, 108.47, 108.30, 104.32, 104.19, 102.13, 100.30, 91.24, 89.70, 97.97, 81.11, 80.43, 66.29, 65.66, 60.45, 58.65, 52.57, 51.67, 51.44, 45.39, 44.44, 37.48, 37.41, 36.42, 34.66, 30.90, 28.01, 26.16, 24.70, 24.66, 24.57, 20.97, 20.39, 20.29, 13.09, 12.73. HRMS-ESI: m/z Calcd for C_27_H_36_N_2_O_7_Na [M+Na]^+^: 523.2415; Found: 523.2394.


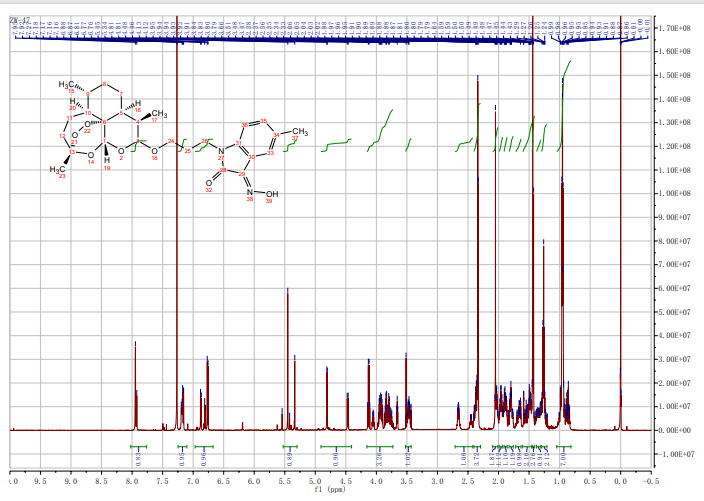


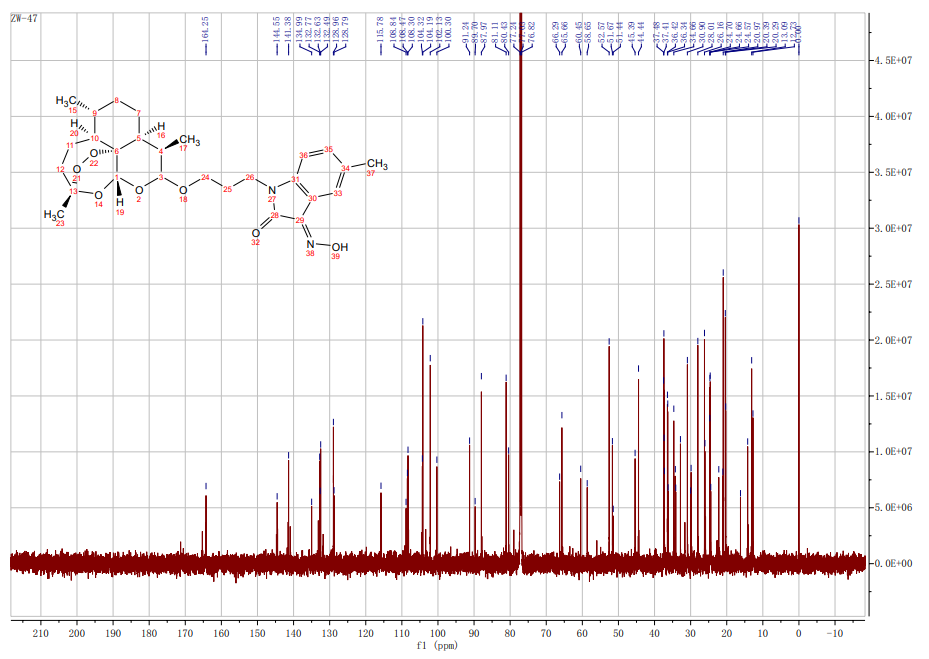


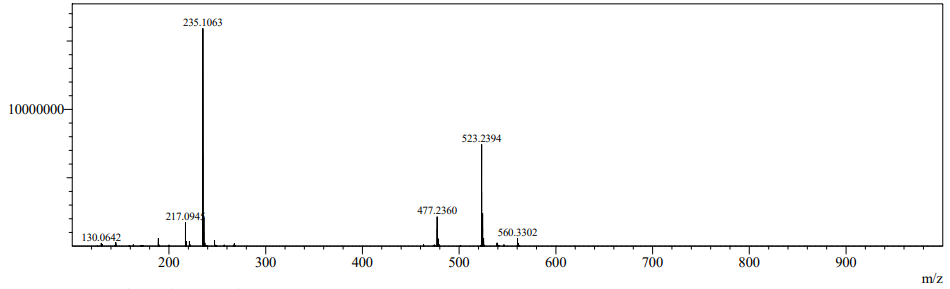


2-(5-methyl-2-oxo-1-(3-(((3*R*,5a*S*,6*R*,8a*S*,9*R*,12*R*,12a*R*)-3,6,9-trimethyldecahydro-12*H*-3,12-epoxy[1,2]dioxepino[4,3-i]isochromen-10-yl)oxy)propyl)indolin-3-ylidene)hydrazine-1-carbothioamide (**6h**)

Yellow solid. ^1^H NMR (600 Hz, CD_3_OD) 0.93-1.02 (m, 7H), 1.23-1.34 (m, 2H), 1.40-1.50 (m, 4H), 1.52-1.59 (m, 1H), 1.65-1.73 (m, 2H), 1.77-1.91 (m, 2H), 1.94-2.06 (m, 3H), 2.34-2.39 (m, 4H), 2.64-2.66 (m, 3H), 3.42-3.49 (m, 1H), 3.73-4.05 (m, 3H), 4.80 (d, *J* = 2.0 Hz, 1H), 5.48 (s, 1H), 6.62 (brs, 1H), 6.80 (d, *J* = 4.0 Hz, 1H), 7.18 (d, *J* = 4.0 Hz, 1H), 7.40 (s, 1H), 7.52 (brs, 1H), 12.88 (s, 1H). ^13^C NMR (150 Hz, DMSO-*d*_6_) 180.01, 161.11, 141.13, 132.91, 132.00, 121.57, 119.44, 109.12, 104.17, 102.17, 87.96, 81.12, 65.34, 52.66, 44.42, 37.35, 37.22, 36.43, 30.90, 27.90, 26.18, 24.69, 24.54, 21.02, 20.47, 13.09, 12.71. HRMS-ESI: m/z Calcd for C_28_H_38_N_4_O_6_SNa [M+Na]^+^: 581.2404; Found: 581.2377.


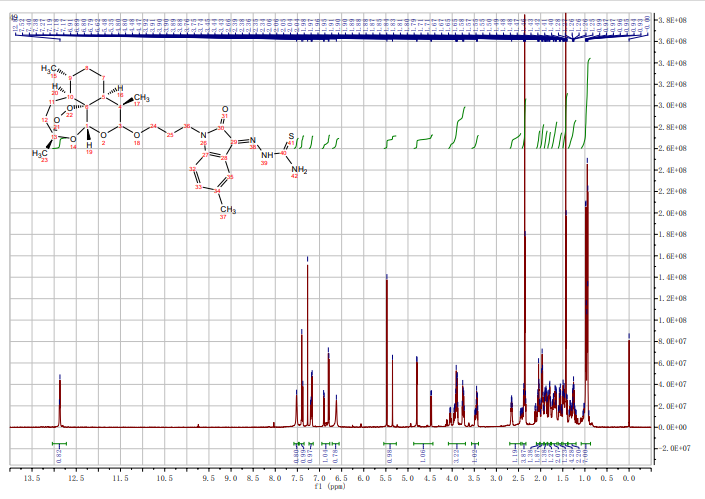


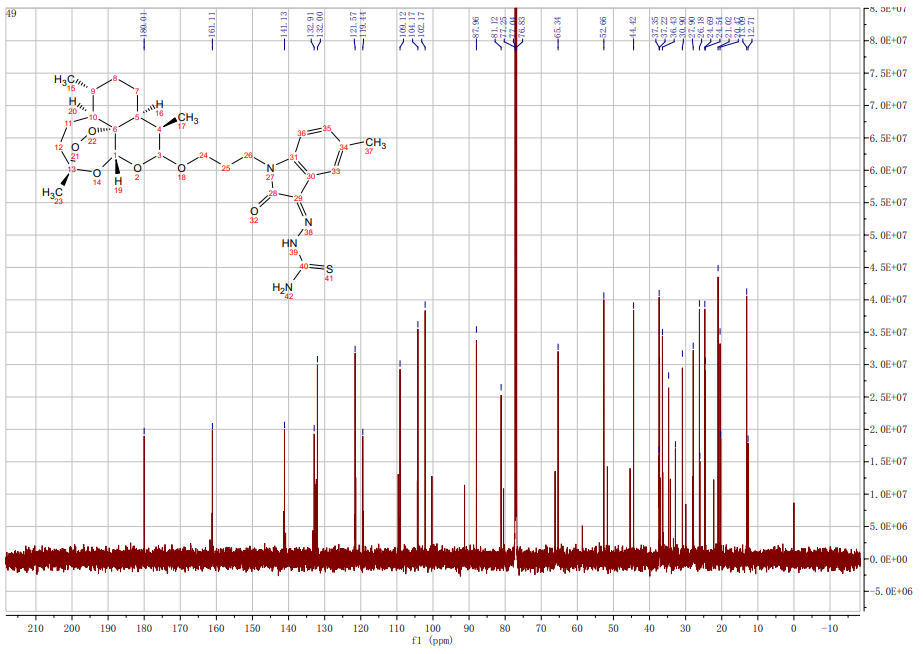


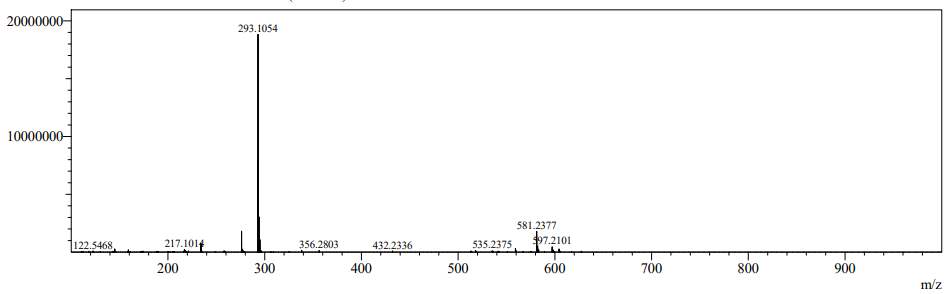


3-(hydroxyimino)-5-methoxy-1-(3-(((3*R*,5a*S*,6*R*,8a*S*,9*R*,12*R*,12a*R*)-3,6,9-trimethyldecahydro-12*H*-3,12-epoxy[1,2]dioxepino[4,3-i]isochromen-10-yl)oxy)propyl)indolin-2-one (**6i**)

Yellow solid. ^1^H NMR (600 Hz, CD_3_OD) 0.76-0.86 (m, 7H), 1.03-1.28 (m, 6H), 1.30-1.40 (m, 1H), 1.48-1.51 (m, 1H), 1.56-1.64 (m, 2H), 1.74-1.94 (m, 4H), 2.17-2.42 (m, 2H), 3.35-3.44 (m, 1H), 3.70 (s, 3H), 3.73-4.02 (m, 3H), 4.60 (d, *J* = 2.0 Hz, 1H), 5.28 (s, 1H), 6.86-6.95 (m, 2H), 7.58 (d, *J* = 2.0 Hz, 1H). ^13^C NMR (150 Hz, CD_3_OD) 168.53, 160.08, 148.01, 140.65, 120.36, 120.33, 117.72, 113.25, 107.90, 106.19, 104.15, 91.80, 84.74, 70.08, 58.85, 56.50, 41.49, 40.99, 39.95, 38.31, 34.86, 31.66, 28.62, 28.33, 28.18, 23.31, 15.93. HRMS-ESI: m/z Calcd for C_27_H_68_N_2_O_8_Na [M+Na]^+^: 539.2364; Found: 539.2354.


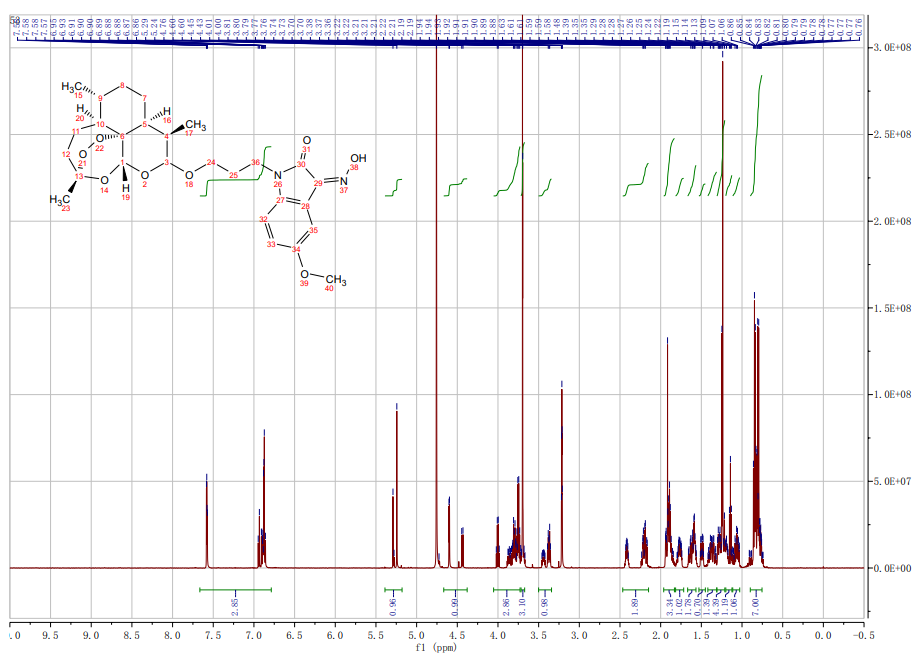


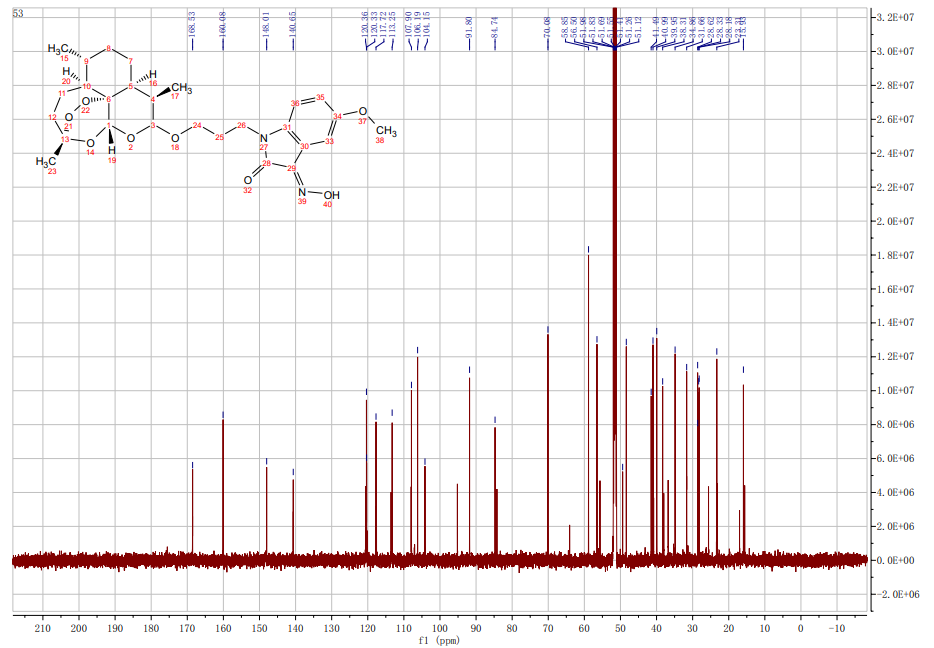


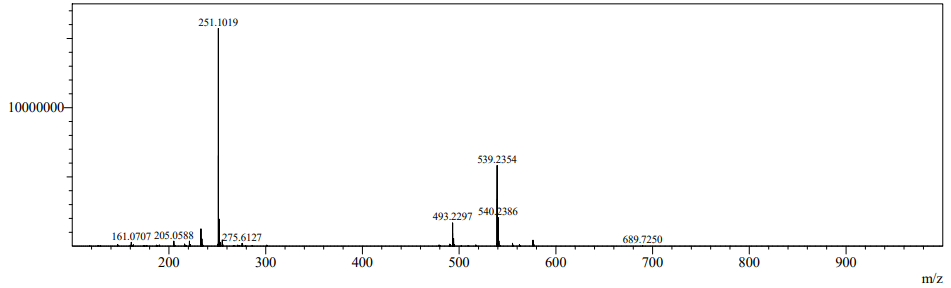


2-(5-methoxy-2-oxo-1-(3-(((3*R*,5a*S*,6*R*,8a*S*,9*R*,12*R*,12a*R*)-3,6,9-trimethyldecahydro-12*H*-3,12-epoxy[1,2]dioxepino[4,3-i]isochromen-10-yl)oxy)propyl)indolin-3-ylidene)hydrazine-1-carbothioamide (**6j**)

Yellow solid. ^1^H NMR (600 Hz, CDCl_3_) 0.86-1.01 (m, 7H), 1.20-1.33 (m, 2H), 1.40-1.55 (m, 5H), 1.58-1.82 (m, 6H), 1.86-1.88 (m, 1H), 2.01-2.05 (m, 1H), 2.33-2.63 (m, 2H), 3.39-3.50 (m, 1H), 3.71-4.02 (m, 6H), 4.76 (d, *J* = 4.0 Hz, 1H), 5.36 (s, 1H), 5.52 (s, 2H), 6.80-6.86 (m, 1H), 7.12-7.18 (m, 2H). ^13^C NMR (150 Hz, CDCl_3_) 178.99, 160.05, 155.30, 136.04, 131.36, 119.28, 116.54, 116.34, 109.05, 105.54, 105.48, 103.29, 103.15, 101.16, 99.29, 90.29, 86.94, 80.10, 65.16, 64.31, 54.95, 51.64, 50.65, 44.37, 43.40, 36.32, 36.21, 35.40, 33.69, 29.87, 26.86, 25.16, 23.66, 23.52, 19.44, 12.07, 11.68. HRMS-ESI: m/z Calcd for C_38_H_37_N_4_O_7_S [M-H]^-^: 573.2388; Found: 573.2363.


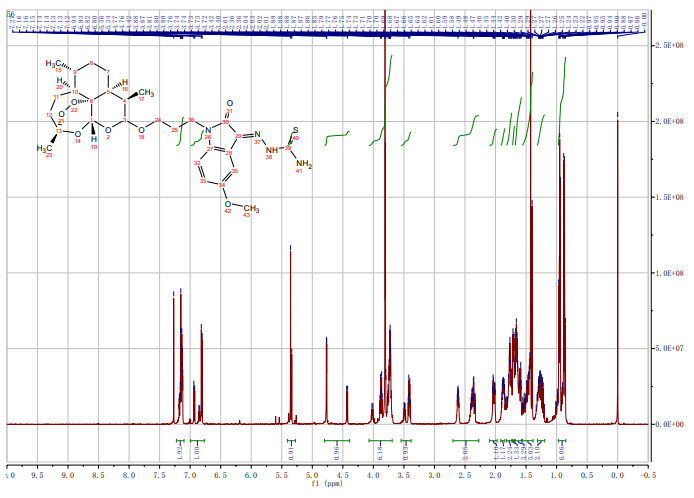


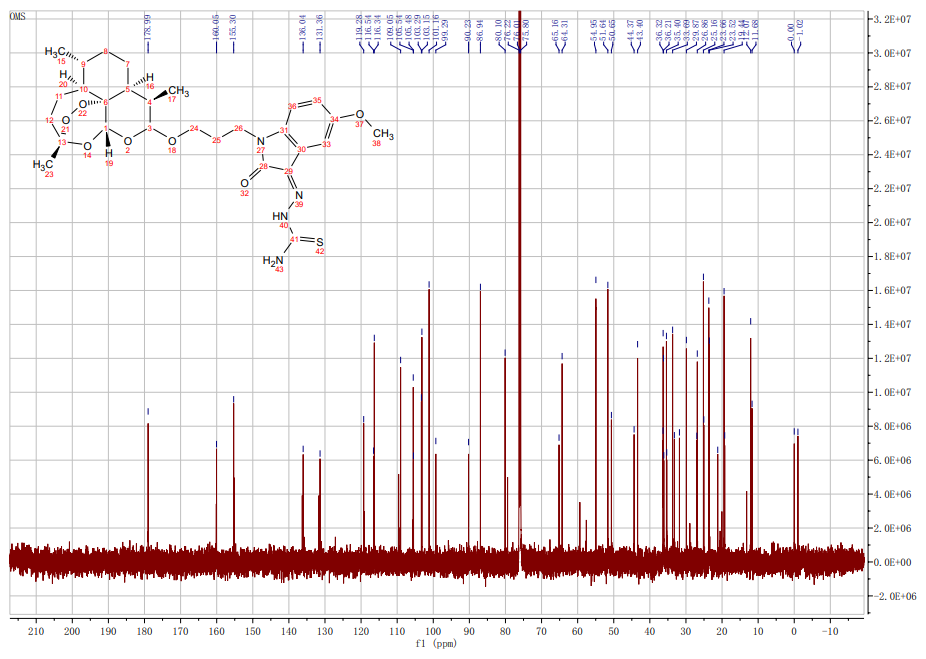


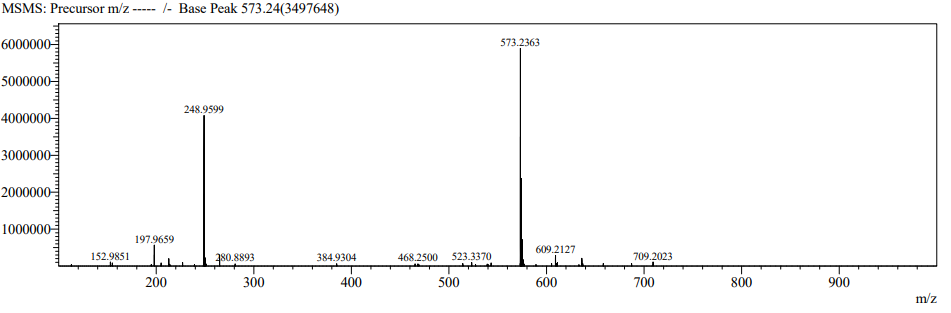


1. ***In vitro* antiproliferative activity evaluation**

A549, A549/DOX, and A549/DDP lung cancer cells (2×10^3^) were plated in each well of a 96-well plate and were allowed to adhere and spread for 24 h. The 1,2,3-triazole tethered dihydroartemisinin-isatin hybrids **8a-c** and **9a-k** were added to a final concentration of 100 *µ*M, and the cells were cultured for 24 h at 37 ^o^C. 3-(4,5-dimethyl-2-thiazolyl)-2,5-diphenyltetrazolium bromide (MTT) solution (10 *µ*L) was added to each well, and the cultures were incubated for an additional 4 h. A further 100 *µ*L of MTT solution was added and incubation continued overnight. The absorbance at 540 nm was determined in each well with a 96-well plate reader. The growth of the treated cells was compared with that of untreated cells.

1. ***In vitro* cytotoxic study**

The cytotoxicity (CC_50_) of the synthesized 1,2,3-triazole tethered dihydroartemisinin-isatin hybrids **8a-c** and **9a-k** were examined by the MTT assay in mouse embryonic fibroblast cells NIH/3T3. The compounds were dissolved in DMSO with concentrations from 1024 to 1 μg/mL. The NIH/3T3 cells were maintained in culture medium at 37 ^o^C under 5% CO_2_ atmosphere. Cells were seeded in 96-well plates (1×104 cell per well) and allowed to recover for 24 h. After 72 h of exposure, cells were harvested and cell viability was assessed by MTT assay. The CC_50_ values were calculated by Bliss analysis.

1. **Pharmacokinetic profiles determination**

CD-1 mice mice (20-25 g) were used in the pharmacokinetic study, and each treatment group had 3 mice which were dosed with hybrids **8a,c** suspension at 30 mg/kg by single intravenous (iv) administration. Compounds were suspended in 0.5% CMC for iv, and blood was collected from the jugular vein of each mouse at the following time points: 0.25, 0.5, 1, 2, 4, 6, 8 and 24 h after oral administration. Total area under the concentration time curve (AUC), the elimination half-time (t_1/2_), the peak concentration (C_max_) and the time to reach peak concentration (T_max_) of samples were determined directly from the experimental data using WinNonlin V6.2.1.
